# Bromodomain protein BRD4 inhibitor JQ1 regulates potential prognostic molecules in advanced renal cell carcinoma

**DOI:** 10.18632/oncotarget.25190

**Published:** 2018-05-01

**Authors:** Takashi Sakaguchi, Hirofumi Yoshino, Satoshi Sugita, Kazutaka Miyamoto, Masaya Yonemori, Yoichi Osako, Makiko Meguro-Horike, Shin-Ichi Horike, Masayuki Nakagawa, Hideki Enokida

**Affiliations:** ^1^ Department of Urology, Graduate School of Medical and Dental Sciences, Kagoshima University, Kagoshima, Japan; ^2^ Division of Functional Genomics, Advanced Science Research Center, Kanazawa University, Kanazawa, Japan

**Keywords:** bromodomain, BRD4, JQ1, sunitinib resistance, renal cell carcinoma

## Abstract

Sunitinib is a standard molecular-targeted drug used as a first-line treatment for metastatic clear cell renal cell carcinoma (ccRCC); however, resistance to sunitinib has become a major problem in medical practice. Recently, bromodomain containing 4 (BRD4), a member of the bromodomain family proteins, was identified as a promising therapeutic target, and its inhibitor JQ1 has been shown to have inhibitory effects in various human cancers. However, the anti-cancer effects of JQ1 in ccRCC, particularly sunitinib-resistant ccRCC, are still unclear. Here, we aimed to elucidate the anti-cancer effects of JQ1 and the mechanisms underlying BRD4 inhibition in sunitinib-sensitive and -resistant ccRCCs. Analysis of The Cancer Genome Atlas (TCGA) ccRCC cohort showed that patients with high *BRD4* expression had shorter overall survival than those with low expression. JQ1 treatment significantly inhibited tumor growth of sunitinib-sensitive and -resistant ccRCC cells in part through MYC regulation. Based on RNA sequencing analyses of ccRCC cells treated with JQ1 to elucidate the mechanisms other than MYC regulation, we identified several oncogenes that may be potential therapeutic targets or prognostic markers; patients with high expression of *SCG5*, *SPOCD1*, *RGS19*, and *ARHGAP22* had poorer overall survival than those with low expression in TCGA ccRCC cohort. Chromatin immunoprecipitation assays revealed that these oncogenes may be promising BRD4 targets, particularly in sunitinib-resistant ccRCC cells. These results identified *SCG5, SPOCD1, RGS19*, and *ARHGAP22* as potential prognostic markers and showed that BRD4 inhibition may have applications as a potential therapeutic approach in sunitinib-sensitive and -resistant ccRCC.

## INTRODUCTION

Molecular targeted drugs inhibiting vascular endothelial growth factor (VEGF) or mammalian target of rapamycin (mTOR) have been widely used for patients with metastatic or recurrent clear cell renal cell carcinoma (ccRCC) [1, 2]. Among these drugs, sunitinib is a common molecular-targeted drug that is recommended as a first-line therapy against advanced ccRCC; indeed, sunitinib treatment has been shown to result in relatively longer progression-free survival and higher response rate [2, 3]. However, sunitinib therapy is not expected to have curative effects because it extends progression-free survival only slightly due to acquisition of resistance to sunitinib [4]. Moreover, metabolism re-programming was observed in RCC cell that showed sunitinib resistance to evade undesirable environment in our previous study [5]. Recently, anti-programmed death-1 (PD-1) antibodies were approved for the treatment of patients with advanced ccRCC. However, the phase ΙΙΙ CheckMate 025 study showed that the objective response rate to anti-PD-1 antibodies was only 25% and that overall survival was improved only slightly [6]. In addition, hypoxia-inducible factor (HIF) 2α antagonists, which are currently under development, have shown excellent anti-cancer effects *in vitro* and *in vivo* and improved progression-free survival in patients with advanced or metastatic ccRCC [7, 8]. Although HIF2α antagonists have promising therapeutic potency, long-term treatment results in acquired resistance through HIF mutations [7]. Therefore, it is necessary to identify new therapeutic approaches to overcome sunitinib resistance.

Bromodomain and extraterminal (BET) family proteins, which includes BRD2, BRD3, BRD4, and BRDT, are epigenetic proteins that interact with acetylated lysine residue on histones to assemble chromatin complexes and transcription activators at specific promoter sites [9, 10]. In many recent studies, BET proteins have been shown to regulate the expression of several important oncogenes (e.g., *MYC*) in several types of cancer [11, 12]. The small-molecule BET inhibitor JQ1 occludes bromodomain acetyl-lysine-binding pockets and is highly specific for BET family proteins, particularly bromodomain containing 4 (BRD4) [9]. Many previous studies have shown that JQ1 induces cytotoxicity in a variety of cancers (e.g., multiple myeloma, leukemia, breast cancer, and prostate cancer) due to binding to BRD4 and preventing its interaction with several oncogenes [10, 13–15]. However, its anti-cancer efficacy has still not been extensively studied in ccRCC, particularly in sunitinib-resistant ccRCC.

Therefore, in this study, we aimed to investigate the anti-cancer efficiency of JQ1 *in vitro* and *in vivo* and to elucidate the molecular mechanisms underlying BRD4 inhibition in sunitinib-sensitive and -resistant ccRCC. First, we investigated the anti-cancer effects of JQ1 *in vitro* and *in vivo* using ccRCC cell lines, including sunitinib-resistant 786-o (SU-R-786-o), which we had previously established *in vivo* [5]. To identify key molecules in sunitinib-resistant ccRCC cells treated with JQ1, we performed RNA sequencing. From this analysis, we found that several oncogenes were significantly downregulated by JQ1 treatment in sunitinib-sensitive and -resistant ccRCC cells and that the expression levels of these genes were significantly associated with cancer progression and survival, according to The Cancer Genome Atlas (TCGA) ccRCC cohort. We also performed chromatin immunoprecipitation (ChIP) assays and found novel and promising BRD4 targets that may contribute to sunitinib resistance in ccRCC.

## RESULTS

### Clinical significance of BRD4 expression in ccRCC

**Figure 1:**
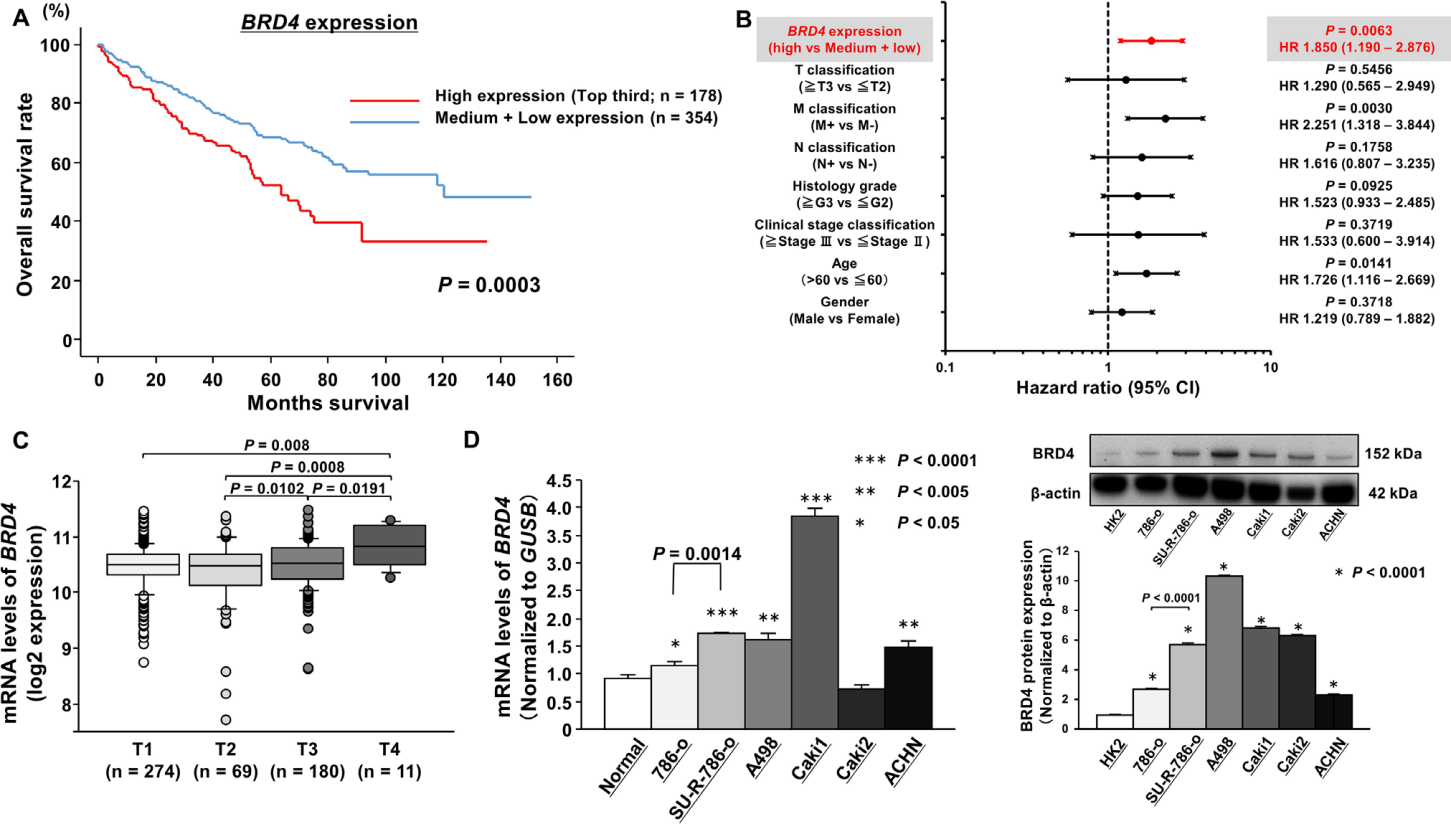
Clinical significance of *BRD4* expression in ccRCC according to TCGA data (**A**) Overall survival (OS) was significantly shortened in patients with high *BRD4* expression (top third) compared with that in patients with medium and low *BRD4* expression (*P* = 0.0003). (**B**) Cox proportional analysis revealed that *BRD4* expression may be an independent predictor for OS (*P* = 0.0063). (**C**) The significant positive correlation between *BRD4* expression and pathological T stage. (**D**) Left; The expression levels of *BRD4* mRNA in RCC cell lines and normal human kidney tissues, as determined by qRT-PCR. *GUSB* was used as an internal control (^***^P < 0.0001, ^**^P < 0.005, ^*^P < 0.05). Right; The expression levels of BRD4 protein in RCC cell lines and human kidney HK2 cell line, as determined by Western blot analyses and densitometry analyses. β-actin was used as a loading control (^*^P < 0.0001).

First, to examine the correlation of *BRD4* expression levels with overall survival (OS), we performed Kaplan-Meier analysis using TCGA database. Among the ccRCC cohort in TCGA, we investigated 532 patients for whom *BRD4* expression and survival time data could be obtained. The cohort was divided into three groups based on the number of patients. As a result, we found that the high *BRD4* expression group (*n* = 178; top third) had significantly lower overall survival rates than patients with low and medium *BRD4* (*n* = 354) expression (*P* = 0.0003, Figure 1A). In addition, when the patients were divided into two groups according to the median *BRD4* expression, the log-rank test showed that overall survival was still significantly shortened in patients with high *BRD4* expression group (*n* = 266) in comparison with low *BRD4* expression group (*n* = 266) (*P* = 0.0044; Supplementary Figure 1A). We also examined the correlation of other bromodomain proteins (*BRD2*, *BRD3*, and *BRDT*) expression with overall survival by Kaplan-Meier analyses. However, there were no significant correlations between *BRD2* or *BRD3* expression and overall survival in TCGA ccRCC cohort (Supplementary Figure 1B, 1C). In terms of *BRDT*, a testis specific bromodomain protein, it was impossible to analyze because of its extremely low expression according to TCGA data. In addition, when the cohort was divided into three groups, there was a significant association between *BRD4* expression and OS after controlling for clinicopathological parameters (i.e., tumor grade, stage, metastasis), age, and sex in a multivariable analysis (*P* = 0.0063, Figure 1B). On the other hand, when the cohort was divided into two groups, the high *BRD4* expression was not significant but tended to be an independent prognostic predictor for OS (*P* = 0.0624, Supplementary Figure 1D). These results suggested that BRD4 may have more oncogenic functions than other bromodomain proteins and higher *BRD4* expression may be a prognostic factor in ccRCC patients. Although there was no significant difference of *BRD4* expression between ccRCC samples and normal samples (Supplementary Figure 2A), we found that the expression level of *BRD4* was significantly increased in advanced T stage cases (Figure 1C, Supplementary Figure 2B). Moreover, we evaluated the expression level of *BRD4* in RCC cell lines by quantitative real-time reverse transcription polymerase chain reaction (qRT-PCR). The expression levels of *BRD4* were significantly upregulated in several RCC cell lines except for Caki2 cells compared with those in normal kidneys (Figure 1D; left). Interestingly, *BRD4* expression in SU-R-786-o cells was significantly upregulated compared with that of 786-o cells (*P* = 0.0014). Furthermore, western blot analyses demonstrated that BRD4 protein expressions in several ccRCC cells were elevated in comparison with the levels in human kidney HK2 cells (Figure 1D; right). Interestingly, BRD4 protein expression in SU-R-786-o cells was also significantly elevated compared with the levels in parent 786-o cells (*P* < 0.0001). These data suggested that BRD4 may be a potential prognostic marker and that treatment by JQ1 as a BRD4 inhibitor may suppress cancer progression and improve survival rates in ccRCC.

### Effects of JQ1 treatment on cell growth in ccRCC cell lines

**Figure 2:**
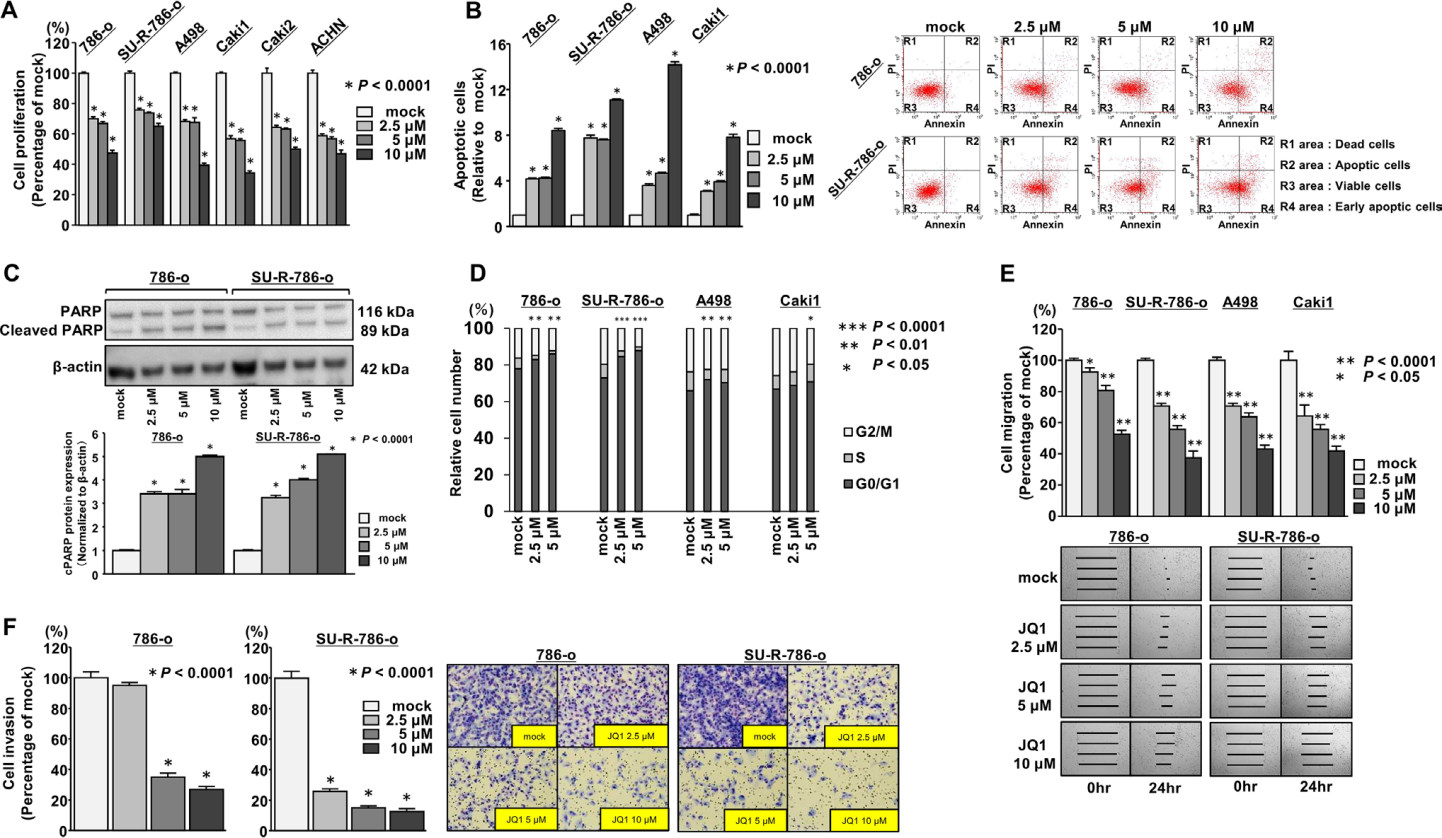
Effects of JQ1 treatment on cell proliferation, migration and invasion in ccRCC cell lines (**A**) Cell proliferation was determined by XTT assays (^*^P < 0.0001). *(***B**) Apoptosis assays were carried out using flow cytometry. Early apoptotic cells are in area R4 and apoptotic cells are in area R2. The normalized ratio of apoptotic cells are shown in the histograms. Cyclohexiamide (2 μg/mL) was used as positive control (^*^P < 0.0001). (**C**) Western blot analyses for apoptotic markers (cleaved PARP) in 786-o and SU-R-786-o cells. β-Actin was used as a loading control. Densitometry analyses using ImageJ software were performed (^*^P < 0.0001). (**D**) Cell cycle assays were carried out using flow cytometry. The bar charts represent the percentage of mock cells in G0/G1, S, and G2/M phases (^***^P < 0.0001, ^**^P < 0.01, ^*^P < 0.05). (**E**) Wound healing assays by JQ1 in ccRCC cell lines. (^**^P < 0.0001, ^*^P < 0.05). (**F**) Matrigel invasion assays by JQ1 in ccRCC cell lines. (^*^P < 0.0001).

We hypothesized that JQ1 may have anti-cancer effects in ccRCC cells. XTT assays revealed significant inhibition of cell proliferation in a concentration-dependent manner in several ccRCC cell lines, including SU-R-786-o cells treated with JQ1 (2.5, 5, and 10 μM), in comparison with the mock treatment (*P* < 0.0001, Figure 2A). Although XTT assays revealed that JQ1 treatment inhibited cell proliferation in six ccRCC cell lines regardless of the expression levels of BRD4 (Figure 2A), A498 and Caki1 cells with relatively high BRD4 expression (Figure 1D) were selected for further analyses in addition to 786-o and SU-R-786-o. Cell apoptosis assays revealed that JQ1 had significant apoptotic effects in several ccRCC cells including SU-R-786-o cells (*P* < 0.0001, Figure 2B, Supplementary Figure 3A). Interestingly, the apoptotic effects in SU-R-786-o cells were comparable to those in other sunitinib-sensitive ccRCC cells (Figure 2B). Western blot analysis showed increased cleaved PARP levels in JQ1- treated- ccRCC cells including SU-R-786-o cells compared with that in mock cells (Figure 2C, Supplementary Figure 3B). We also investigated the cell cycle progression using 786-o, SU-R-786-o, A498, and Caki1 cells treated with JQ1. The fraction of cells in the G0/G1 phase was significantly higher in ccRCC cells treated with JQ1 (2.5, or 5 μM) compared with that in mock cells (Figure 2D). These data suggested that JQ1 may have anti-cancer effects on cancer cell growth in ccRCC, including sunitinib-resistant ccRCC.

### Effects of JQ1 treatment on cell migration and invasion in ccRCC cell lines

Next, we investigated whether JQ1 treatment suppressed cancer cell migration and invasion activity in ccRCC cells. Wound healing assays demonstrated that cell migration activity was significantly inhibited in a concentration-dependent manner in ccRCC cell lines, including SU-R-786-o cells treated with JQ1 (2.5, 5, and 10 μM) in comparison with that in mock cells (Figure 2E). Additionally, Matrigel invasion assays demonstrated that the number of invading cells was significantly decreased in a concentration-dependent manner in 786-o and SU-R-786-o cells treated with JQ1 compared with that in mock cells (Figure 2F), although we could not find significant inhibition of invasion activities in A498 and Caki1 cells treated with JQ1 (Supplementary Figure 3C). These data suggested that JQ1 may suppress cancer progression and metastasis in ccRCC, including sunitinib-resistant ccRCC.

### Effects of JQ1 treatment on SU-R-786-o xenograft tumor growth *in vivo*

**Figure 3:**
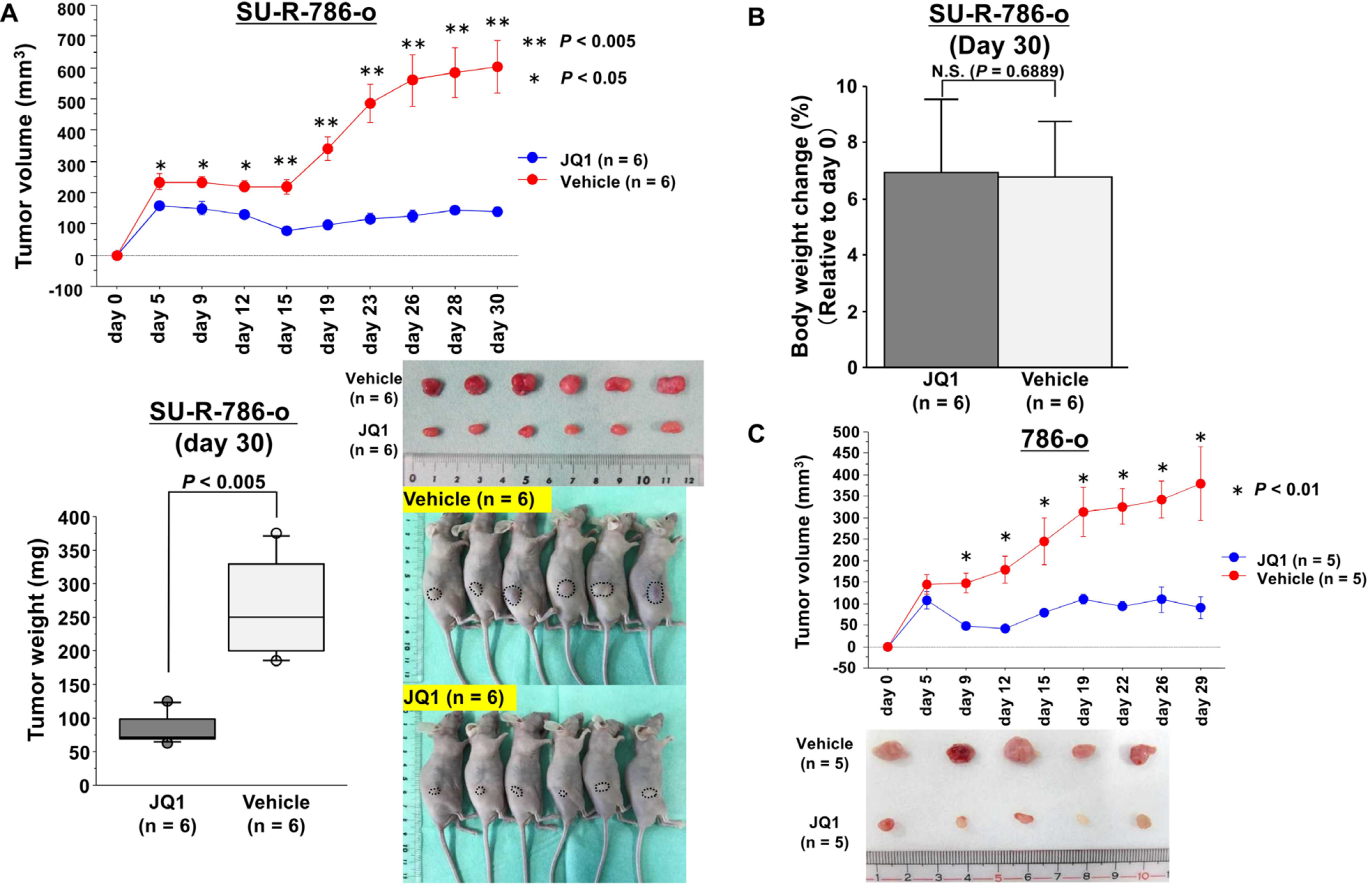
Effects of JQ1 treatment on SU-R-786-o xenograft tumor growth *in vivo* (**A**) Xenograft assays using SU-R-786-o cells revealed that JQ1 treatment significantly suppressed tumor growth in comparison with vehicle treatment (^**^P < 0.005, ^*^P < 0.05). (**B**) There were no significant differences in mouse weights between the JQ1-treated group and vehicle-treated group. (**C**) Xenograft assays using 786-o cells demonstrated that tumor growth was significantly suppressed in mice treated with JQ1 compared with that in vehicle-treated mice (*P* < 0.01).

To determine the anti-cancer effects of JQ1 treatment in sunitinib-resistant ccRCC cells *in vivo*, we performed xenograft assays using SU-R-786-o cells. We injected SU-R-786-o cells subcutaneously into nude mouse and successfully established a xenograft mouse model. We found that tumor growth was significantly suppressed in mice treated with JQ1 compared with that in vehicle-treated mice (*P* < 0.005, Figure 3A). In addition, there were no significant differences in body weights between the JQ1-treated group and vehicle-treated group (Figure 3B). We also performed xenograft assays using 786-o cells and found a similar therapeutic effect (*P* < 0.01, Figure 3C). These results strongly supported that JQ1 has anticancer effects without causing major adverse events in sunitinib-sensitive and - resistant ccRCC.

### JQ1 treatment inhibited MYC expression in ccRCC cells

**Figure 4:**
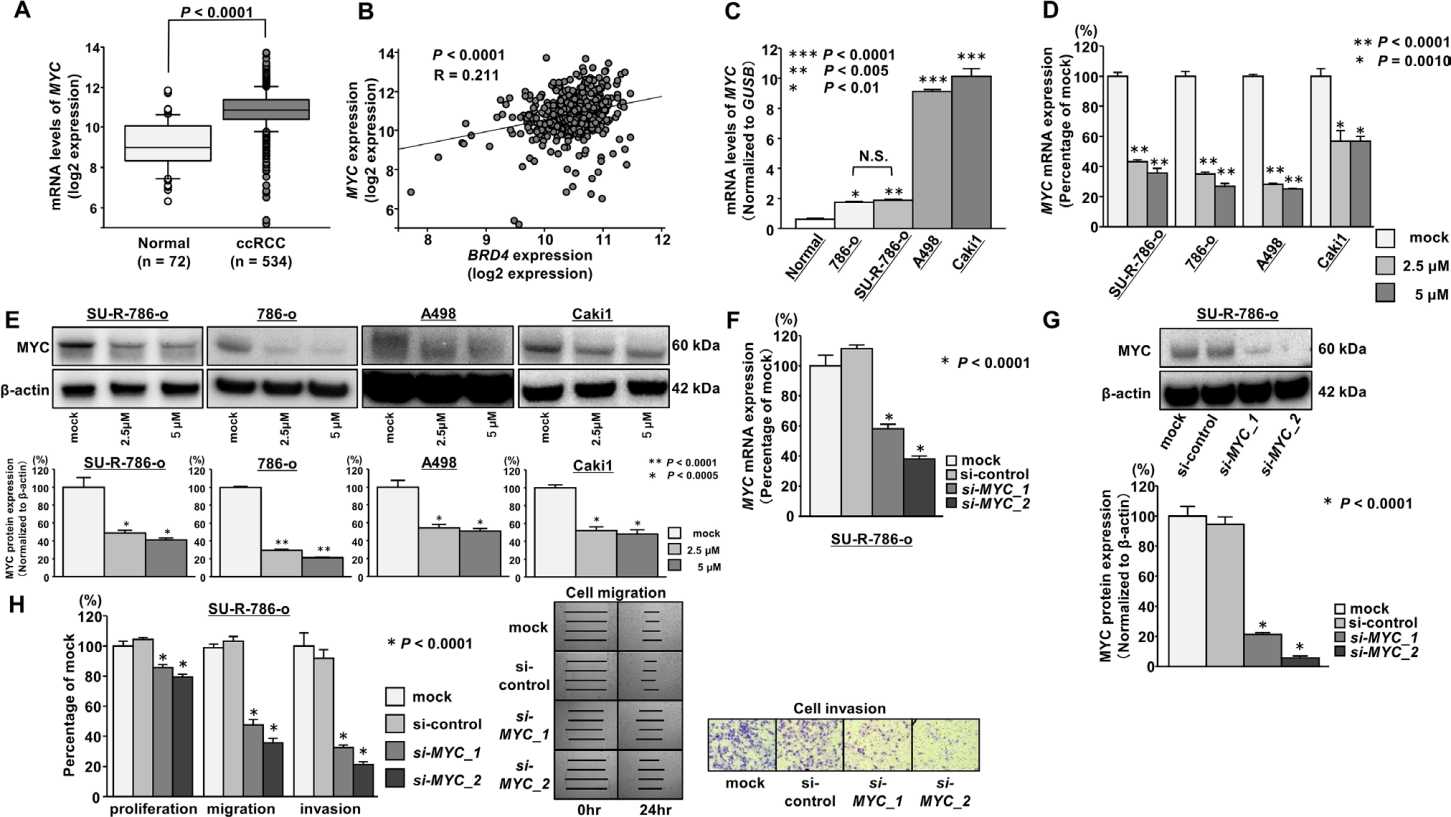
JQ1 treatment inhibited MYC expression in ccRCC cells (**A**) The expression level of *MYC* mRNA was significantly upregulated in ccRCC tissues compared with that in normal kidney tissues according to TCGA data (*P* < 0.0001). (**B**) Positive correlation between *BRD4* and *MYC* expression in ccRCC tissues according to TCGA data (*P* < 0.0001, *R* = 0.211). (**C**) *MYC* mRNA was significantly upregulated in ccRCC cell lines compared with that in normal kidneys, as determined by qRT-PCR (^***^P < 0.0001, ^**^P < 0.005, ^*^P < 0.01). (**D**) The expression of *MYC* mRNA was significantly repressed in JQ1-treated ccRCC cells (2.5 and 5 μM) in comparison with that in mock cells. *GUSB* was used as an internal control (^*^P < 0.0001). (**E**) The expression of MYC protein was markedly repressed in JQ1-treated ccRCC cells (2.5 and 5 μM) in comparison with that in mock cells. β-Actin was used as a loading control. Densitometry analyses using ImageJ software were performed (^**^P < 0.0001, ^*^P < 0.0005). (**F**) The expression of *MYC* mRNA was significantly repressed in si-*MYC* transfectants in comparison with that in mock or si-control transfectants. *GUSB* was used as an internal control (^*^P < 0.0001). (**G**) The expression of MYC protein was markedly repressed in si-*MYC* transfectants in comparison with mock or si-control transfectants. β-Actin was used as a loading control. Densitometry analyses using ImageJ software were performed (*P* < 0.0001). (**H**) Cell proliferation, migration, and invasion activities were significantly inhibited in si-*MYC* transfectants in comparison with that in mock or si-control transfectants (^*^ *P* < 0.0001).

Several previous studies demonstrated that BRD4 could regulate *MYC* gene transcription and that JQ1 effectively suppresses cancer cell proliferation by inhibiting BET-mediated regulation of *MYC* in various types of cancer [13, 14, 16, 17]. Additionally, we previously demonstrated the significance of the oncogene *MYC* in RCC [18]. Thus, we investigated whether JQ1 treatment suppressed *MYC* expression in ccRCC cells, particularly in SU-R-786-o cells. Among the ccRCC cohort in TCGA, the expression level of *MYC* was significantly upregulated in patients with ccRCC (*n* = 534) compared with that in normal patients (*n* = 72; *P* < 0.0001; Figure 4A). Specifically, there was a significant positive correlation between *MYC* and *BRD4* expression (*P* < 0.0001, R = 0.211; Figure 4B). Moreover, *MYC* mRNA expression in ccRCC cells was significantly upregulated compared with that in normal kidneys (Figure 4C). qRT-PCR showed that JQ1 treatment (2.5 or 5 μM) significantly downregulated *MYC* mRNA expression in ccRCC cell lines, including SU-R-786-o cells (Figure 4D). In addition, western blot analysis showed that MYC protein expression was reduced in JQ1-treated RCC cells including SU-R-786-o (Figure 4E). To investigate the functional role of *MYC* in SU-R-786-o cells, we performed loss-of-function studies using cells transfected with two *si-MYC* constructs. We found satisfactory knockdown of *si-MYC* transfection in SU-R-786-o cells by qRT-PCR and western blot analysis (Figure 4F, 4G). Cell proliferation, migration, and invasion were inhibited by *si-MYC* transfection in SU-R-786-o cells compared with that in mock or control cells (Figure 4H). These results suggested that the anti-cancer effects of JQ1 treatment in sunitinib-resistant ccRCC cells were induced in part through BET-mediated inhibition of *MYC*.

### Identification of novel molecular targets of JQ1 treatment in sunitinib-resistant ccRCC

**Figure 5:**
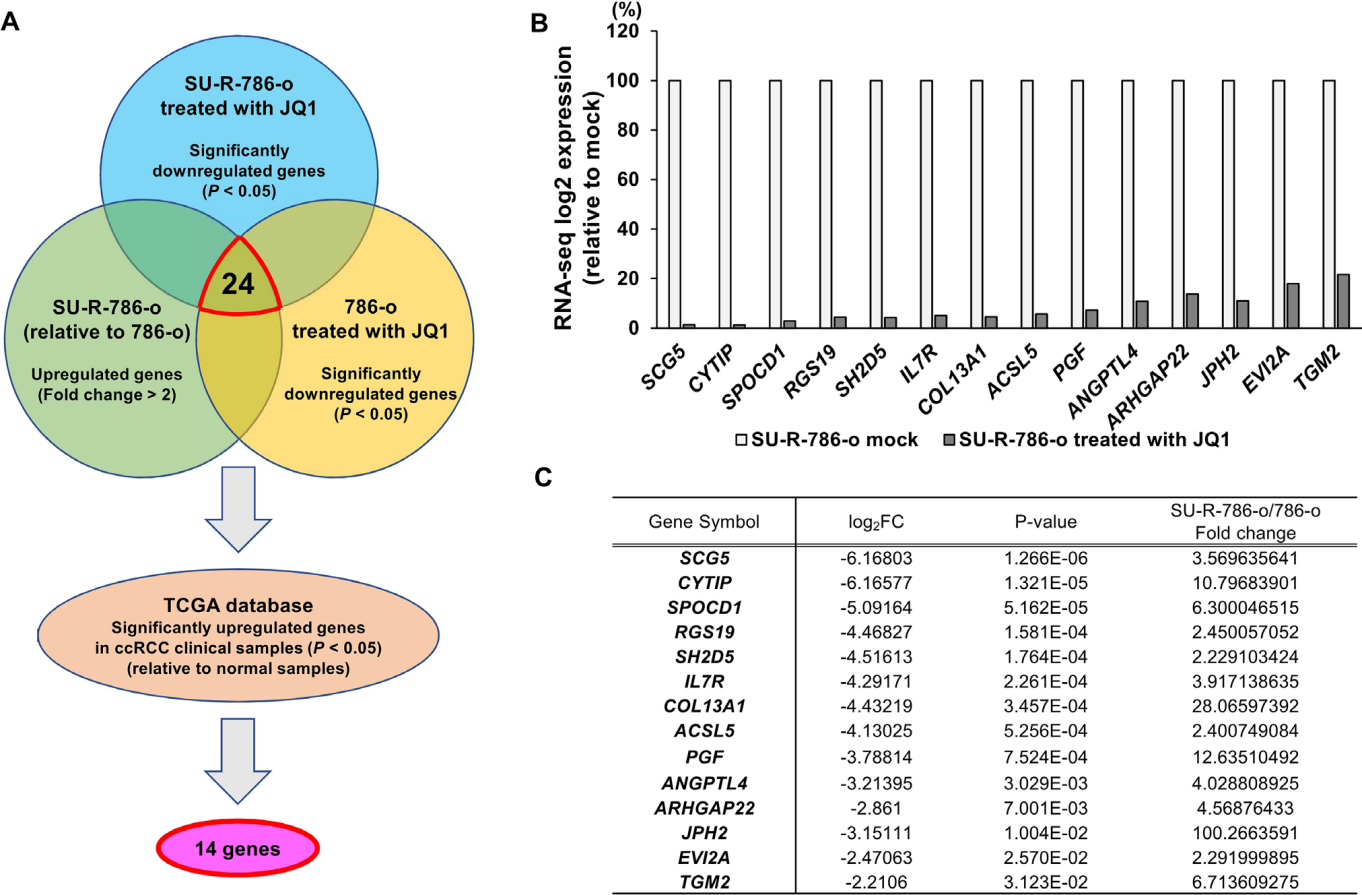
Identification of novel molecular targets and mechanisms by JQ1 treatment in sunitinib-resistant ccRCC (**A**) The strategy for analysis of target genes regulated by JQ1 treatment in sunitinib-resistant ccRCC. (**B**–**C**) Fourteen genes were negatively regulated by JQ1 treatment in RNA-seq analysis of SU-R-786-o cells.

As described above, we demonstrated the anti-cancer effects of JQ1 in sunitinib-sensitive and -resistant ccRCC cells. Furthermore, downregulation of MYC by JQ1 treatment was found to play a partial role in the anti-cancer effects of sunitinib-resistant ccRCC. However, the elevation of *MYC* expression in SU-R-786-o cells was not dramatically increased in comparison with other sunitinib-sensitive ccRCC cells, and there were no significant changes in *MYC* expression between SU-R-786-o and 786-o cells (Figure 4C). Moreover, we found no significant relationships between the clinicopathological parameters (i.e., tumor stage, grade, survival rate) and the expression level of *MYC* in TCGA ccRCC cohort (data not shown). Therefore, we hypothesized that there may be other prior and MYC-independent molecular mechanisms and target genes regulated by JQ1 treatment in sunitinib-resistant ccRCC. First, to gain further insights into the molecular mechanisms regulated by JQ1 treatment in sunitinib-resistant ccRCC, we performed RNA sequencing of JQ1-treated- or untreated- SU-R-786-o and 786-o cells. Figure 5A shows our strategy to narrow down the target genes of JQ1 treatment. Based on the RNA sequencing data, we found 525 genes significantly downregulated in JQ1 (2.5 μM)- treated- SU-R-786-o cells. Among these genes, we also found 24 genes that were significantly upregulated in SU-R-786-o cells in comparison with 786-o cells and downregulated by JQ1 treatment (2.5 μM) in 786-o cells. Moreover, among these 24 genes, we identified 14 genes that were significantly upregulated in TCGA ccRCC clinical samples (*n* = 534) in comparison with normal samples (*n* = 72; Supplementary Figure 4). We speculated that these genes may be associated with sunitinib resistance and could have some clinical significance in ccRCC. Finally, we focused on these 14 genes as oncogenes regulated by JQ1 and performed further analysis of these genes (Figure 5B, 5C). In addition, to improve our knowledge of the molecular mechanisms regulated by JQ1 treatment in sunitinib-resistant ccRCC, we also performed pathway analysis using Gene set enrichment analysis (GSEA). We analyzed 942 genes that were significantly upregulated or downregulated by JQ1 treatment in SU-R-786-o cells (Supplementary Figure 5A). Interestingly, among the 14 genes mentioned above, 9 genes were implicated in at least one of the top 20 enriched pathways (Supplementary Figure 5B). Furthermore, among these 20 enriched pathways, several pathways associated with G-protein coupled receptor (GPCR) signaling were highly ranked within this list (Supplementary Figure 5C). Notably, there were no pathways associated with MYC-dependent signaling within this list. Collectively, these results suggested that there may be novel MYC- independent molecular mechanisms and oncogenes regulated by JQ1 treatment in sunitinib-resistant ccRCC.

### Clinical significance of 14 genes in ccRCC

**Figure 6:**
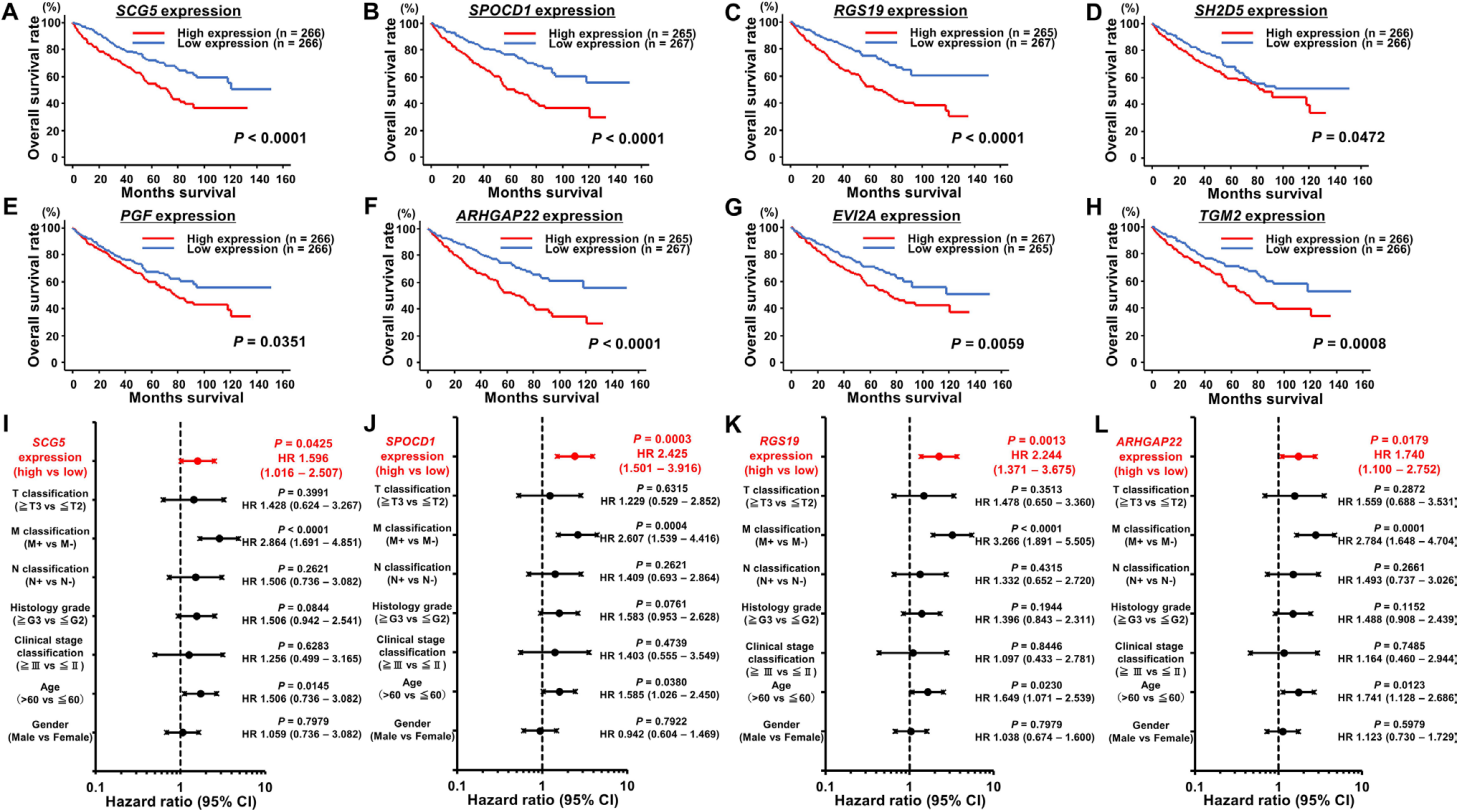
Kaplan-Meier survival plots for overall survival in TCGA ccRCC cohort (**A**–**H**) For 8 genes (*SCG5*, *SPOCD1*, *RGS19*, *SH2D5*, *PGF*, *ARHGAP22*, *EVI2A*, and *TGM2*), overall survival was significantly reduced in patients with high mRNA expression in comparison with that in patients with low expression in ccRCC. (**I**–**L**) Cox proportional analysis showed that *SCG5*, *SPOCD1*, *RGS19*, and *ARHGAP22* were independent predictors of overall survival in ccRCC.

**Figure 7:**
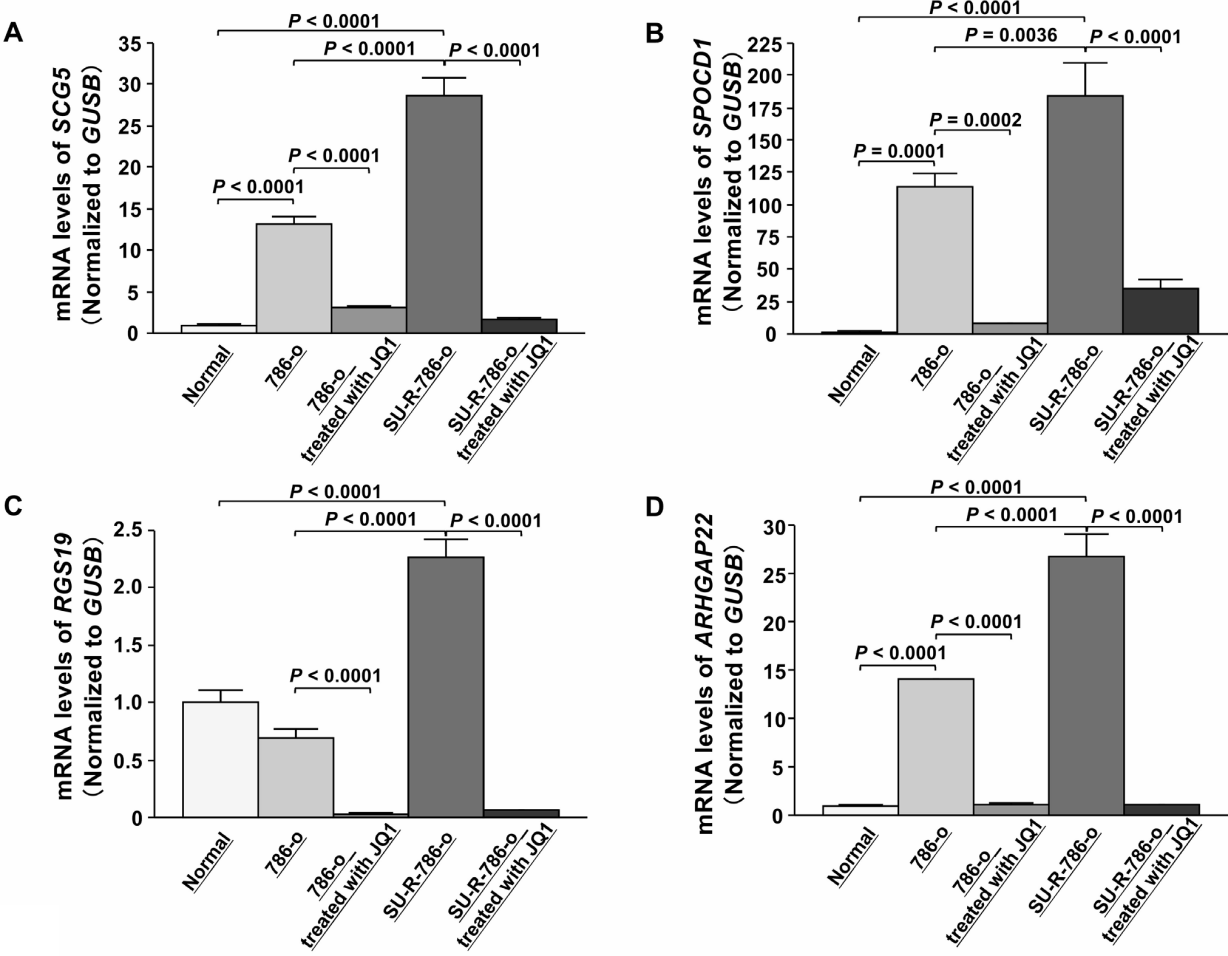
Effects of JQ1 treatment on the expression of *SCG5, SPOCD1, RGS19,* and *ARHGAP22* mRNAs in 786-o and SU-R-786-o cells (**A**–**D**) qRT-PCR demonstrated that *SCG5*, *SPOCD1*, *RGS19*, and *ARHGAP22* were significantly upregulated in SU-R-786-o cells compared with those in 786-o cells and downregulated by JQ1 treatment (2.5 μM) in 786-o and SU-R-786-o cells. *GUSB* was used as an internal control.

**Figure 8:**
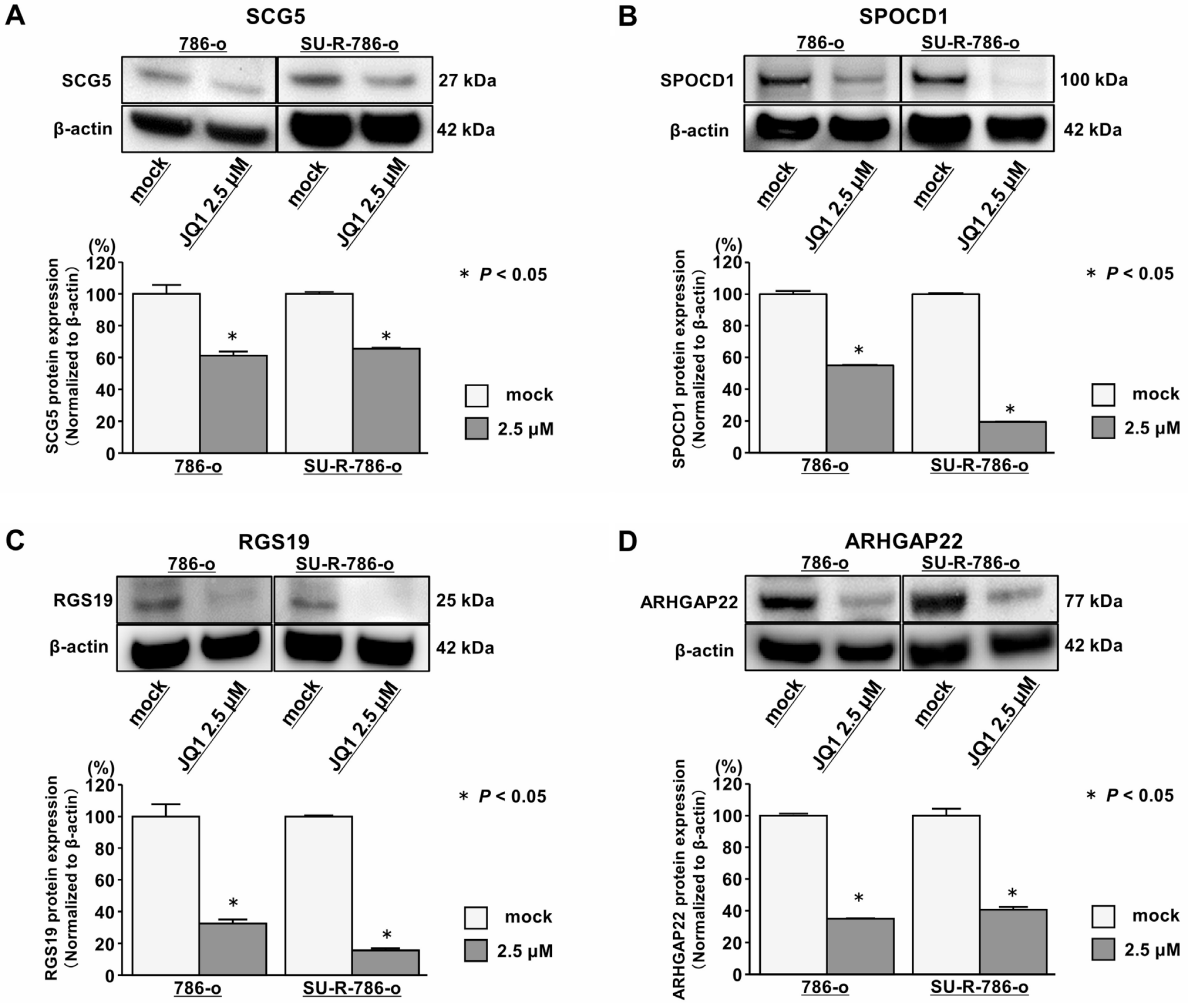
Effects of JQ1 treatment on the expression of SCG5, SPOCD1, RGS19, and ARHGAP22 proteins in 786-o and SU-R-786-o cells (**A**–**D**) Western blot analyses demonstrated that *SCG5*, *SPOCD1*, *RGS19*, and *ARHGAP22* were repressed by JQ1 treatment (2.5 μM) in 786-o and SU-R-786-o cells. β-actin was used as an internal control. Densitometry analyses using ImageJ software were performed (*P* < 0.05).

To determine the clinical significance of these 14 genes, we investigated the association between the expression levels of these genes and clinicopathological parameters in TCGA ccRCC samples. Among these 14 genes, the expression levels of 12 genes (excluding *IL7R* and *ANGPTL4*) were significantly related to some clinicopathological parameters (Supplementary Figures 6–17). These data suggested that JQ1 treatment may contribute to suppression of ccRCC progression through inhibition of specific gene expression. Additionally, we performed Kaplan-Meier analyses to examine the correlation between the expression levels of these genes and disease-free survival (DFS) in TCGA ccRCC samples. Among the ccRCC cohort in TCGA, we applied 534 patients for whom mRNA expression of these genes could be obtained, and divided them into two groups based on median value. In addition, among these patients, we investigated 432 patients for whom disease-free survival time data could be obtained. As the results, high expression levels of 7 genes (*SCG5*, *SPOCD1*, *RGS19*, *PGF*, *ARHGAP22*, *JPH2*, and *TGM2*) were significantly associated with poorer DFS rates (Supplementary Figure 18A–18G). Multivariate analysis demonstrated that *SCG5*, *SPOCD1*, *JPH2*, and *TGM2* were independent DFS predictors (Supplementary Figure 18H–18K). Furthermore, we performed Kaplan-Meier analyses to examine the correlation between the expression levels of these genes and OS in TCGA ccRCC samples. Among the ccRCC cohort in TCGA, we applied 534 patients for whom mRNA expression of these genes could be obtained, and divided them into two groups based on median value. In addition, among these patients, we investigated 532 patients for whom OS time data could be obtained. As the results, high expression levels of eight genes (*SCG5*, *SPOCD1*, *RGS19*, *SH2D5*, *PGF*, *ARHGAP22*, *EVI2A*, and *TGM2*) were significantly associated with poorer OS (Figure 6A–6H). Multivariate analysis demonstrated that *SCG5*, *SPOCD1*, *RGS19*, and *ARHGAP22* were independent OS predictors (Figure 6I–6L). qRT-PCR demonstrated that the expression levels of these 4 genes were significantly upregulated in SU-R-786-o cells compared with those in 786-o cells and downregulated by JQ1 treatment (2.5 μM) in 786-o and SU-R-786-o cells (Figure 7A–7D). In addition, western blot analysis showed that the protein expression levels of these four genes were reduced in JQ1-treated 786-o and SU-R-786-o cells (Figure 8A–8D). Collectively, these data suggested that JQ1 treatment may contribute to improving the survival rates (DFS and OS) of patients with sunitinib-sensitive and -resistant ccRCC through comprehensive regulation of the expression levels of these genes.

### *SCG5*, *SPOCD1*, *RGS19*, and *ARHGAP22* were directly targeted by BRD4 through binding to the specific gene promoters in sunitinib-resistant 786-o cells

**Figure 9:**
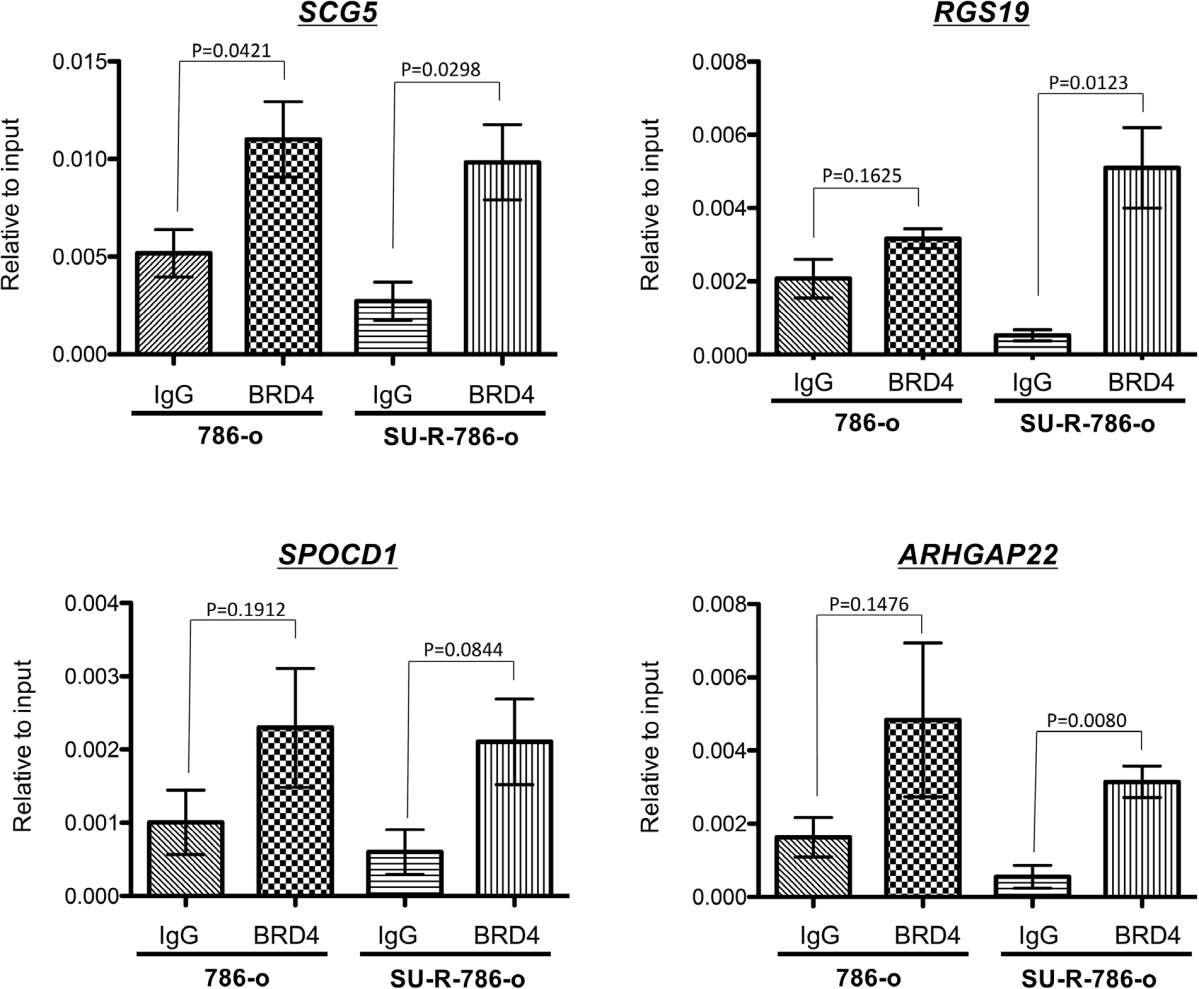
BRD4 bound to the promoter regions of *SCG5, SPOCD1, RGS19,* and *ARHGAP22* genes ChIP assays were performed using normal rabbit IgG or anti-BRD4 antibodies as described in the Materials and Methods. We found that BRD4 was highly enriched at promoter regions of *SCG5*, *SPOCD1*, *RGS19*, and *ARHGAP22* in SU-R-786-o cells.

Our data demonstrated that *SCG5*, *SPOCD1*, *RGS19*, and *ARHGAP22* were novel OS predictors and may play major roles in ccRCC. Finally, to determine whether BRD4 bound at promoter regions of these genes, we performed ChIP assays using anti-BRD4 antibodies. BRD4 was highly enriched at the proximal promoter regions of *SCG5*, *SPOCD1*, *RGS19*, and *ARHGAP22* in SU-R-786-o cells (*P* = 0.0298, *P* = 0.0844, *P* = 0.0123, and *P* = 0.0080, respectively; Figure 9). Interestingly, BRD4 recruitment at promoter regions of these genes in SU-R-786-o cells was increased compared with that in 786-o cells (Figure 9), suggesting that BRD4 recruitment at these promoters may contribute to sunitinib resistance through promoting the transcription of these oncogenes. Taken together, these results strongly supported the excellent anti-cancer effects of BRD4 inhibition by JQ1 in sunitinib-resistant ccRCC and suggested that *SCG5*, *SPOCD1*, *RGS19*, and *ARHGAP22* may be novel direct targets of the epigenetic reader BRD4 in sunitinib-resistant ccRCC.

## DISCUSSION

Among several BET inhibitors, JQ1 is a noteworthy anticancer drug that is able to permeate the cell membrane and has a structure that mimics acetyl-lysine, allowing it to bind to the acetyl-lysine pocket possessed by bromodomain proteins [12]. Although JQ1 also inhibits other bromodomain proteins such as BRD2 and BRD3, it has been reported that JQ1 is more specific to BRD4 than other bromodomain proteins by several previous studies [12, 13, 19]. In addition, among these proteins, several recent studies have revealed that BRD4 plays crucial roles in transcription programs induced by cancer [19–22]. Our Kaplan-Meier analyses also showed that *BRD4* expression was significantly correlated with overall survival, whereas *BRD2* and *BRD3* expression were showed no correlation based on the TCGA ccRCC cohort. Interestingly, several recent studies have reported that a highly selective BRD4 inhibitor which induces BRD4 protein degradation has been developed [23, 24]. Therefore, further studies are needed to investigate the anti-cancer effects of its highly selective BRD4 inhibition in sunitinib-sensitive and -resistant ccRCC.

The anticancer effects of JQ1 are mediated by MYC down-regulation in many types of cancer [12, 13]. Zhu et al. reported that JQ1 inhibits thyroid tumor growth in a mouse model by decreasing MYC abundance and attenuating cyclin D1-CDK4-Rb-E2F3 signaling [17]. Additionally, Bian et al. showed that MYC-high patient-derived xenografts were highly sensitive to JQ1 treatment in pancreatic cancer [16]. However, in recent studies, the MYC-independent mechanisms of JQ1 have been reported in several cancers [15, 25–27]. Wang et al. reported that JQ1 radiosensitizes non-small cell lung cancer cells by upregulating p21 [25]. Additionally, several recent studies have reported that BRD4 inhibition suppresses the transcription of programmed death-ligand 1 (PD-L1) independently from MYC regulation [26, 27]. In our present study, we found that JQ1 had anti-cancer effects through MYC regulation in ccRCC cells; however, these effects were observed regardless of innate MYC expression. Thus, we hypothesized that JQ1 may have MYC-independent mechanisms, and subsequently performed RNA-seq analysis with 786-o parent and SU-R-786-o cells, which showed moderate MYC expression.

Based on our RNA-seq analysis, we found 4 novel genes (*SCG5*, *SPOCD1*, *RGS19*, and *ARHGAP22*) which were significantly downregulated in JQ1-treated-cells and may be promising and independent predictors for overall survival in patients with ccRCC. Moreover, ChIP assays demonstrated that these oncogenes may be novel BRD4 targets in sunitinib-resistant ccRCC. Secretogranin V (*SCG5*) is a member of the chromogranin and secretogranin family and is expressed in neuroendocrine tumors, functioning in neuroendocrine differentiation in cancer [28]. Several recent studies have revealed that *SCG5* may be involved in colorectal cancer tumorigenesis by affecting proliferation [29, 30]. SPOC domain containing 1 (*SPOCD1*) encodes a protein belonging to the TF II S family of transcription factors [31]. Meng et al. revealed that *SPOCD1* is associated with gastric cancer risk and carcinogenesis [32]. Interestingly, Antonie et al. reported that *SPOCD1* may predict progression in T1G3 bladder cancer [33]. Regulator of G-protein signaling 19 (*RGS19*) is a member of the RGS family, acting as a multifunctional GTPase-activation protein by regulating GPCR signaling [34]. *RGS19* overexpression has been shown to promote cell proliferation through deregulating cell cycle control and enhancing Akt signaling in several types of cancer [35]. Importantly, GPCRs have recently been shown to play crucial roles in tumor growth and metastasis [36]. Notably, GSEA analysis in our present study suggested that JQ1 treatment may affect GPCR signaling in SU-R-786-o cells. Rho GTPase activating protein 22 (*ARHGAP22*) encodes a member of the GTPase activating protein family which activates a GTPase belonging to the RAS superfamily of small GTP-binding proteins. *ARHGAP22* is activated by Rho-kinase signaling and is responsible for cancer cell movement [37].

The other genes we identified by RNA-seq analysis also play crucial roles in ccRCC. For example, Vanharanta et al. previously revealed that cytohesin 1 interacting protein (*CYTIP*) is an important mediator of the metastatic phenotype driven by the altered VHL-HIF response in ccRCC [38]. They also indicated that DNA demethylation enables HIF-driven CYTIP expression to protect cancer cells from death cytokine signals. In addition, placental growth factor (*PGF*) is a VEGF homolog that exclusively binds VEGF receptor 1 (VEGFR1) and co-receptor neuropilin-1. Bessho et al. reported that inhibition of *PGF* may be effective for the treatment of VEGFR1-expressing tumors by inducing the activity of tumor-associated macrophages for angiogenesis escape in sunitinib-resistant ccRCC [39]. Moreover, we previously reported that silencing of transglutaminase 2 (*TGM2*) inhibited cancer cell proliferation and invasion and that *TGM2* may function as an oncogene in ccRCC [40]. Taken together, our present data suggested that BRD4 inhibition by JQ1 in patients with sunitinib-sensitive and -resistant ccRCC may improve their prognosis and suppress cancer progression by comprehensively suppressing the expression of these oncogenes. However, the function roles of these genes in sunitinib-resistant ccRCC are still unclear. Therefore, further studies are needed to determine the oncogenic functions of these genes in ccRCC, particularly sunitinib-resistant ccRCC.

In our present study, although we found high BRD4 enrichment at *SCG5*, *SPOCD1*, *RGS19*, and *ARHGAP22* promoter regions in SU-R-786-o cells and *SCG5* in 786-o parent cells, we only found a tendency to BRD4 enrichment at the promoter regions of *SPOCD1*, *RGS19*, and *ARHGAP22* in 786-o parent cells. Moreover, the mRNA expression levels of these 4 genes were significantly upregulated in SU-R-786-o cells in comparison with 786-o parent cells. We speculated that the transcription of these 4 genes may have been enhanced in SU-R-786-o cells compared with that in 786-o cells by increasing BRD4 recruitment at these promoters and that these 4 genes may subsequently contribute to sunitinib resistance. Therefore, these differences may be caused by genetic or epigenetic changes through acquisition of sunitinib resistance, as we had previously observed by metabolic reprograming in sunitinib-resistant cells [5]. Moreover, resistance to JQ1 itself has also been demonstrated through *SPOP* mutation, which contributes to resistance to BET inhibitors through BRD4 stabilization in prostate cancer [41–43]. Thus, further studies are needed to elucidate the genetic or epigenetic mechanisms associated with BRD4 during the acquisition of molecular drug resistance.

In conclusion, BRD4 expression may be a potential prognostic marker in ccRCC, and JQ1, as a BRD4 inhibitor, significantly inhibited cancer cell aggressiveness both *in vitro* and *in vivo* in sunitinib-sensitive and -resistant ccRCC. To the best of our knowledge, this is the first report indicating that JQ1 showed anti-cancer effects through downregulation of *SCG5, SPOCD1, RGS19,* and *ARHGAP22*. We also found that these genes were good independent predictors for OS in patients with ccRCC. The discovery of molecular targets mediated by BRD4 inhibition provides important insights into the potential mechanisms of sunitinib resistance, new therapeutics, and novel biomarkers in ccRCC.

## MATERIALS AND METHODS

### Human RCC cell lines and cell culture

Human RCC cell lines 786-o, A498, ACHN, Caki1, and Caki2 and human kidney cortex/proximal tubule epithelial cell line HK2, were obtained from the American Type Culture Collection (ATCC; Manassas, VA, USA). The SU-R-786-o cell line was established by performing gavage feeding of sunitinib (40 or 25 mg/kg, five times a week; Biorbyt, CA, USA) in mice as previously described [5]. Human RCC cell lines were grown in RPMI 1640 medium (Invitrogen; Carlsbad, CA, USA) supplemented with 10% fetal bovine serum (FBS). HK2 cell line was grown in Keratinocyte Serum Free Medium (Invitrogen) supplemented with 0.05 mg/mL bovine pituitary extract (BPE) and 5 ng/mL epidermal growth factor (EGF). These cell lines were maintained in humidified incubators (5% CO_2_) at 37°C. Routine tests for mycoplasma infection were negative.

### Cell proliferation, cell apoptosis, and cell cycle assays

(+)-JQ1 (#4499; Tocris, Bristol, UK) was used as a BET inhibitor. Cell proliferation was determined with XTT assays (Roche Applied Science, Tokyo, Japan) according to the manufacturer’s instructions. Cell cycle assays and cell apoptosis assays were carried out by flow cytometry (CytoFLEX Analyzer; Beckman Coulter, Brea, CA, USA) using a FITC Annexin V Apoptosis Detection Kit (BD Biosciences, Bedford, MA, USA) and Cycletest PLUS DNA Reagent Kit (BD Biosciences) according to the manufacturer’s recommendations as previously described [44].

### Cell migration and invasion assays

Cell migration activity was evaluated with wound healing assays. Cell invasion assays were performed using modified Boyden chambers consisting of Transwell precoated Matrigel membrane filter inserts with 8-μm pores in 24-well tissue culture plates (BD Biosciences). The experimental procedures were performed as described in our previous study [45].

### *In vivo* tumor xenograft model

A mixture containing 100 µL SU-R-786-o cells (5 × 10^6^ cells) or 786-o cells (3 × 10^6^ cells) and 100 µL Matrigel Matrix (Corning, Bedford, MA, USA) was injected subcutaneously into the flanks of female nude mice (BALB/c nu/nu, 6- to 8-weeks-old). (+)-JQ1 (HY-13030; MCE, NJ, USA) was prepared in a vehicle of 10% dimethylsulfoxide and 10% hydroxypropyl-β-cyclodextrin (Sigma, St. Louis, MO, USA) solution and injected at 50 mg/kg intraperitoneally once daily, beginning the day after tumor cell inoculation. The dose was adjusted according to the weight of each mouse, and the volume of injection did not exceed 150 μL. All animal experiments were approved by the animal care review board of Kagoshima University.

### RNA extraction and qRT-PCR

Total RNA was isolated using Isogen (Nippon Gene, Tokyo, Japan) according to the manufacturer’s protocol. We applied a SYBR-Green quantitative PCR-based array approach. qRT-PCR was performed with 500 ng total RNA using Power SYBR Green Master Mix (cat. no. 4367659; Applied Biosystems, Foster City, CA, USA) on a 7300 Real-time PCR System (Applied Biosystems). The specificity of amplification was monitored using the dissociation curve of the amplified product. All data values were normalized to *GUSB*, and the ΔCt method was employed to calculate the fold-change. As normal kidney RNA, we used human kidney total RNA (AM7976; Thermo Fisher Scientific). The following primers were used:

*BRD4*, forward primer, 5′-ACCTCCAACCCTAACAAGCC-3′ and reverse primer, 5′-TTTCCATAGTGTCTTGAGCACC-3′; *MYC*, forward primer, 5′-GGCTCCTGGCAAAAGGTCA-3′ and reverse primer, 5′-CTGCGTAGTTGTGCTGATGT-3′; *SCG5*, forward primer, 5′-ATGCTATCTGGCCTACTGTTTTG-3′ and reverse primer, 5′-GGCCCACAAGATTCATGGC-3′; *SPOCD1*, forward primer, 5′-GGTGCCTACTCAGGGGAGAG-3′ and reverse primer, 5′-CTGGGGCAACTGTCATCTAAG-3′; *RGS19*, forward primer, 5′- GGCGCAGTCTTTTGACAAGC -3′ and reverseprimer, 5′-GCCTTCTCGTCTACCACATGC-3′; *ARHGAP22*, forward primer, 5′-GGCAGCGCCTAGAGGAAAC-3′ and reverse primer, 5′-CACGTCTGTTGTGCTGTCAAA-3′; and *GUSB*, forward primer, 5′-CGTCCCACCTAGAATCTGCT-3′ and reverse primer, 5′-TTGCTCACAAAGGTCACAGG-3′.

### Transfection with small interfering RNA (siRNA)

As described elsewhere [18], RCC cells were transfected with Lipofectamine RNAiMAX transfection reagent (Thermo Fisher Scientific) and Opti-MEM (Thermo Fisher Scientific) with 10 nM siRNA. *MYC* siRNA (SASI_ Hs01_00222676 and SASI_ Hs01_00222677; Sigma-Aldrich) and negative-control siRNA (D-001810-10; Thermo Fisher Scientific) were used in loss-of-function experiments.

### RNA sequencing analyses

Total RNAs from 786-o cells and SU-R-786-o cells were subjected to RNA sequencing, which was performed by Eurofins Japan. mRNA profiles were generated by single-read deep sequencing using Illumina HiSeq 2500/2000.

### Western blotting

Protein lysates were separated on NuPAGE 4–12% Bis-tris gels (Invitrogen) and transferred to polyvinylidene difluoride membranes. Immunoblotting was carried out with diluted anti-BRD4 antibodies (1:1000, ab128874; abcam, Cambridge, UK), anti-MYC antibodies (1:150, #5605; Cell Signaling Technology, Danvers, MA, USA), anti-poly-A ribose polymerase (PARP) antibodies (1:500, #9542; Cell Signaling Technology), anti-cleaved PARP antibodies (1:500, #5625; Cell Signaling Technology), anti-SCG5 antibodies (1:200, 10761-1-AP; proteintech, Chicago, IL, USA), anti-SPOCD1 antibodies (1:1000, 22243-1-AP; proteintech), anti-RGS19 antibodies (1:100, bs-3867R; Bioss, Woburn, MA, USA), anti-ARHGAP22 antibodies (1:500, 3018002; Novus Biologicals, Littleton, CO, USA) and anti-β-actin antibodies (1:5000, bs-0061R; Bioss). Specific complexes were visualized using an echochemiluminescence (ECL) detection system (GE Healthcare, Little Chalfont, UK) as described previously [46]. The expression level of these genes was evaluated using ImageJ software (ver. 1.48; http://rsbweb.nih.gov/ij/index.html) as described previously [18, 40].

### ChIP assays

ChIP assays were performed using ChIP reagent (Nippon Gene, Tokyo, Japan) according to the manufacturer’s protocol with modification, as previously described [47]. Cells were fixed with 1% formaldehyde for 10 min at 37°C. The fixed cells were lysed with SDS lysis buffer and sonicated using Bioruptor UCD-250. Immunoprecipitation was conducted overnight using an antibody specific to BRD4 (#13440; Cell Signaling Technology) or normal rabbit IgG (#2729; Cell Signaling Technology) conjugated to Dynabeads M-280 Sheep anti-mouse IgG (#DB11201; Veritas, Tokyo, Japan), respectively. After washing, the beads were incubated overnight in the ChIP direct elution buffer with proteinase K (20 mg/ml) for 6 h at 65°C for reverse cross-linking. The immune-precipitated DNA was purified using AMPure XP beads (Beckman Coulter) according to the manufacturer’s protocol. The amount of DNA immunoprecipitated by the antibody was determined by qPCR. (KAPA SYBR FAST qPCR Master Mix kit, NIPPON Genetics) The quantitated values of ChIP DNAs were normalized using the percent input method, in which the value for the ChIP DNA is divided by that of an input sample (5% of starting chromatin) and shown as % input. ChIP primer sequences are available upon request.

### Bioinformatics analysis

TCGA cohort database for 534 patients with ccRCC (KIRC) was used for analysis of clinical relevance. Full sequencing information and clinical information were acquired using UCSC Xena (http://xena.ucsc.edu/), cBioPortal (http://www.cbioportal.org/public-portal/), and TCGA (https://tcga-data.nci.nih.gov/tcga/). This study meets the publication guidelines provided by TCGA (http://cancergenome.nih.gov/publications/publicationguidelines). GSEA was performed to identify enriched pathways using open source software v2.0 (www.broad.mit.edu).

### Statistical analysis

The relationships between two groups were analyzed using Mann-Whitney *U* tests. The relationships between three variables and numerical values were analyzed using Bonferroni-adjusted Mann-Whitney *U* tests. Spearman’s rank tests were used to evaluate the correlations between two variables. The overall survival of patients with ccRCC from TCGA cohort was evaluated by the Kaplan-Meier method. Patients were divided into two or three groups based on the number of patients in the cohort, and differences between the two groups were evaluated by log-rank tests. Multivariable analysis was evaluated by the Cox proportional hazard model. All analyses were carried out using Expert StatView software, version 5.0 (Cary, NC, USA). All experiments were performed in triplicate.

## SUPPLEMENTARY MATERIALS FIGURES


