# Supplementary material for: Bromodomain protein BRD4 inhibitor JQ1 regulates potential prognostic molecules in advanced renal cell carcinoma
Source: Oncotarget. 2018 May 1;9(33):23003–17. doi: 10.18632/oncotarget.25190 (PMC5955408; doi:10.18632/oncotarget.25190)
Supplement: Supplementary file 1 [file oncotarget-09-23003-s001.pdf]

Bromodomain protein BRD4 inhibitor JQ1 regulates potential prognostic molecules in advanced renal cell carcinoma

SUPPLEMENTARY MATERIALS

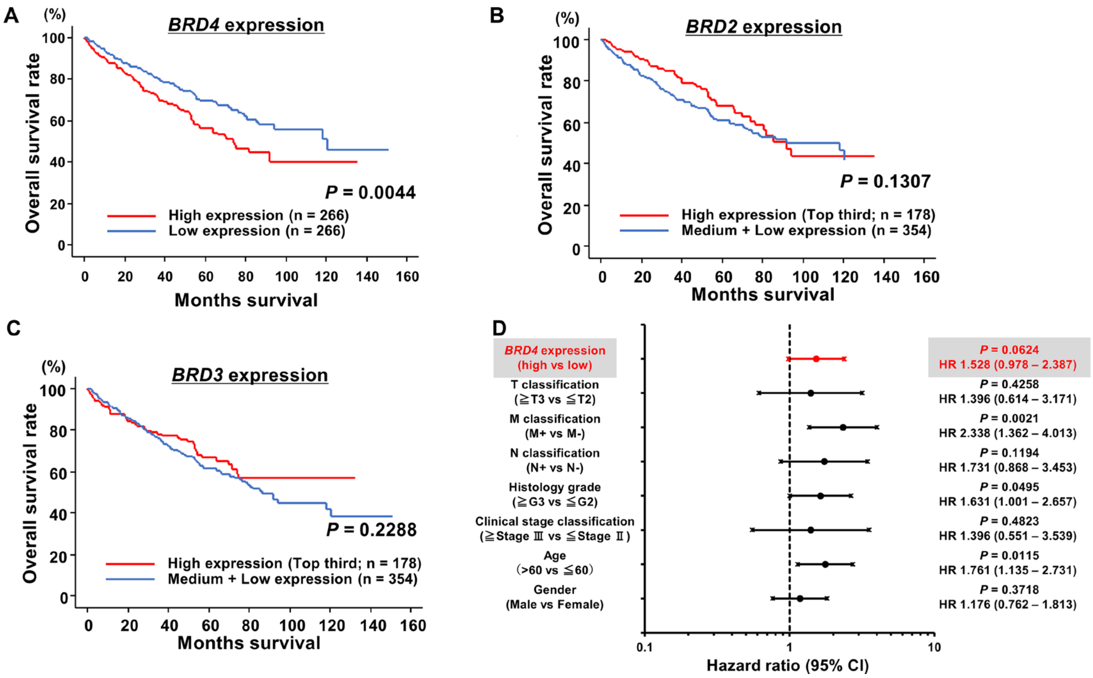

Supplementary Figure 1: Correlations between bromodomain proteins and overall survival in TCGA database. (**A**) High *BRD4* expression group (n = 266) had significantly lower overall survival rates than patients with low *BRD4* (n = 266) expression (P = 0.0044). TCGA ccRCC cohort was divided into two groups based on median value. (**B**–**C**) There were no significant correlations between high *BRD2* or *BRD3* expression and overall survival rates in TCGA ccRCC cohort. (**D**) When the cohort was divided into two groups, the high *BRD4* expression was not significantly independent but tended to be a prognostic predictor for OS in cox hazard proportional model (P = 0.0624).

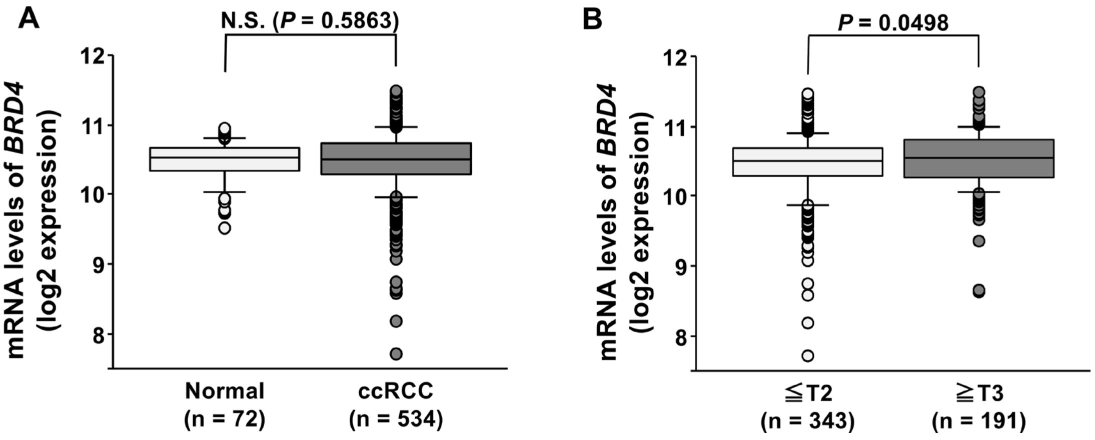

Supplementary Figure 2: *BRD4* mRNA expression in ccRCC samples and normal samples in TCGA database. (**A**) There were no significant differences in *BRD4* expression between ccRCC samples and normal samples. (**B**) The significant positive correlation between *BRD4* expression and pathological T stage.

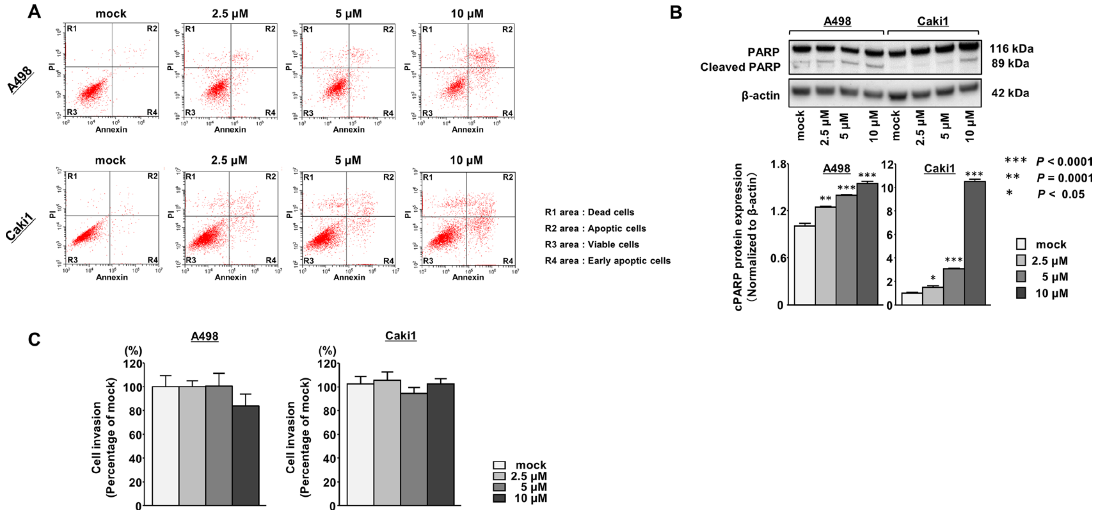

Supplementary Figure 3: Cell apoptosis and Matrigel invasion assays in JQ1- treated A498 and Caki1 cells. (**A**) Apoptosis assays were carried out using flow cytometry. Early apoptotic cells are in area R4 and apoptotic cells are in area R2. (**B**) Western blot analyses for apoptotic markers (cleaved PARP) in A498 and Caki1 cells. β-Actin was used as a loading control. Densitometry analyses using ImageJ software were performed ($^{***}P < 0.0001$, $^{**}P = 0.0001$, $^{*}P < 0.05$). (**C**) Matrigel invasion assays by JQ1 in A498 and Caki1 cells.

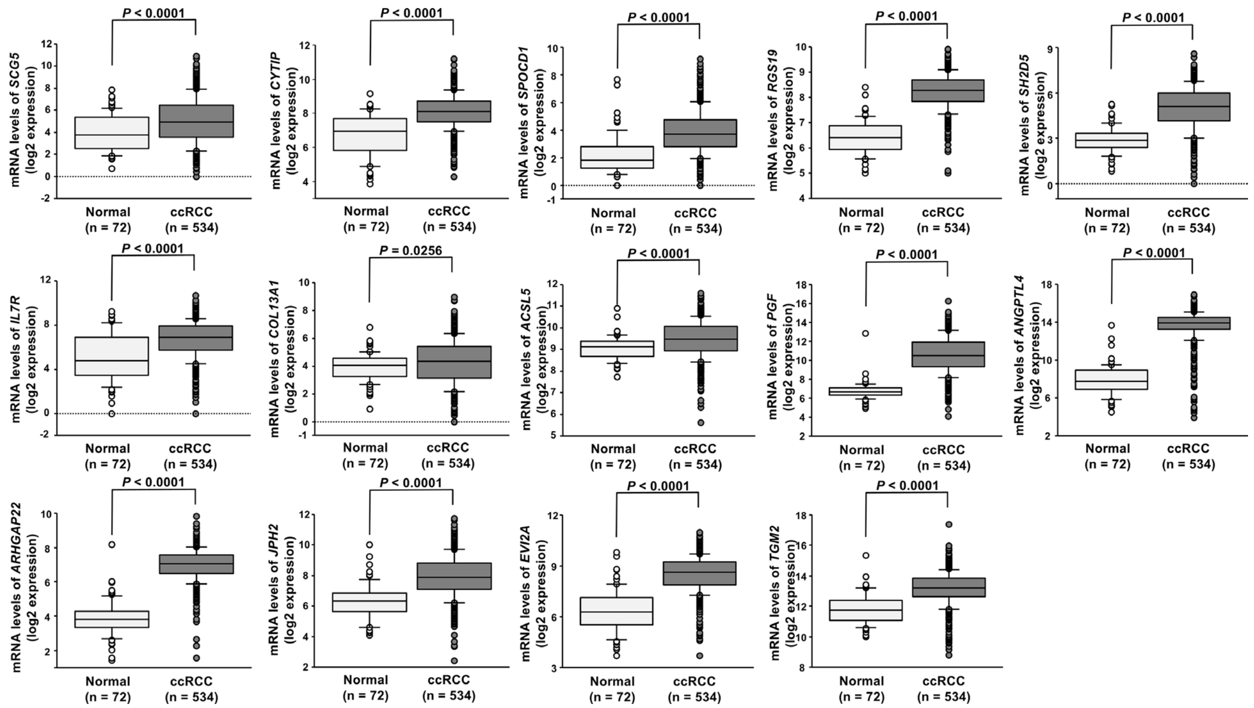

Supplementary Figure 4: mRNA expressions of 14 genes in TCGA ccRCC samples. mRNA levels of 14 genes were significantly upregulated in ccRCC samples in comparison with those in normal samples.

A

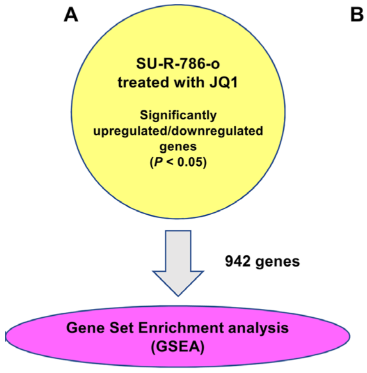

B

NAME	NES (Normalized enrichment score)	Nominal p-value	FDR q-value	Remarks
GO_G_PROTEIN_COUPLED_RECEPTOR_ACTIVITY	-2.5241818	0	0	
NABA_SECRETED_FACTORS	-2.0601797	0	0.035213258	including *PGF* and *ANGPTL4*
GO_REGULATION_OF_HORMONE_SECRETION	-2.0441248	0	0.028435294	including *SCG5*
GO_G_PROTEIN_COUPLED_RECEPTOR_SIGNALING_PATHWAY	-1.8707492	0	0.13375947	including *SCG5* and *RGS19*
REACTOME_SIGNALING_BY_GPCR	-1.7989211	0	0.2079951	including *RGS19*
GO_REGULATION_OF_HORMONE_LEVELS	-1.7867813	1.05E-03	0.19681327	including *SCG5*
GO_NEGATIVE_REGULATION_OF_PHOSPHORUS_METABOLIC_PROCESS	-1.7780328	0	0.18387267	including *SPOCD1*
GO_NEGATIVE_REGULATION_OF_CATALYTIC_ACTIVITY	-1.7671976	0	0.17738593	including *SCG5*, *SPOCD1* and *ANGPTL4*
MIKKELSEN_MEF_HCP_WITH_H3K27ME3	-1.7462083	1.02E-03	0.19287388	
MARTENS_TRETINOIN_RESPONSE_UP	-1.7379339	0	0.18689556	including *SH2D5*, *ANGPTL4* and *TGM2*
REACTOME_GPCR_DOWNSTREAM_SIGNALING	-1.7278335	4.11E-03	0.18714902	including *RGS19*
GO_GROWTH_FACTOR_ACTIVITY	-1.6958995	9.66E-03	0.22564483	including *PGF*
SENESE_HDAC1_AND_HDAC2_TARGETS_UP	-1.694415	2.05E-03	0.21072908	including *SCG5*, *IL7R* and *PGF*
GO_ENZYME_INHIBITOR_ACTIVITY	-1.6925522	1.17E-02	0.19907601	including *SCG5* and *ANGPTL4*
LEE_BMP2_TARGETS_DN	-1.638249	2.01E-03	0.29551452	
KIM_WT1_TARGETS_UP	-1.6360383	1.49E-02	0.28238085	
MEISSNER_NPC_HCP_WITH_H3K4ME2	-1.6352745	0	0.2669951	including *PGF*
GO_NEGATIVE_REGULATION_OF_MOLECULAR_FUNCTION	-1.6310741	0	0.2613682	including *SCG5*, *SPOCD1* and *ANGPTL4*
GO_MACROMOLECULE_CATABOLIC_PROCESS	-1.6301	9.28E-03	0.2501703	
GO_NEGATIVE_REGULATION_OF_PHOSPHORYLATION	-1.6274703	1.66E-02	0.24327084	

C

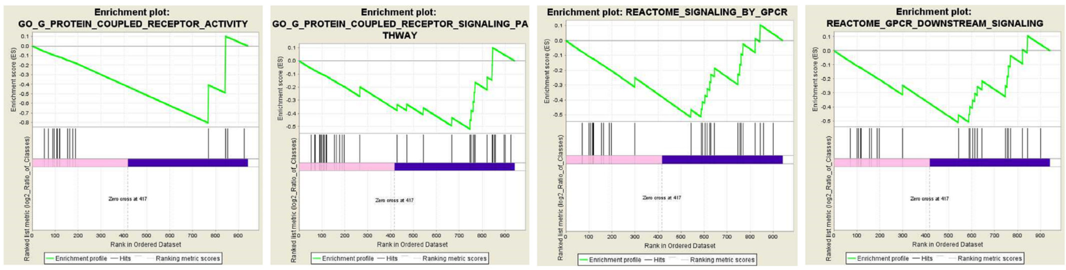

Supplementary Figure 5: Molecular signaling pathways regulated by JQ1 treatment in SU-R-786-o cells were classified by GSEA analysis. (**A**) The strategy for analysis of molecular signaling pathways regulated by JQ1 treatment in SU-R-786-o cells. (**B**) Top 20 enriched pathways indicated by GSEA. (**C**) Several pathways associated with G-protein coupled receptor (GPCR) signaling were highly ranked within the enriched pathways list.

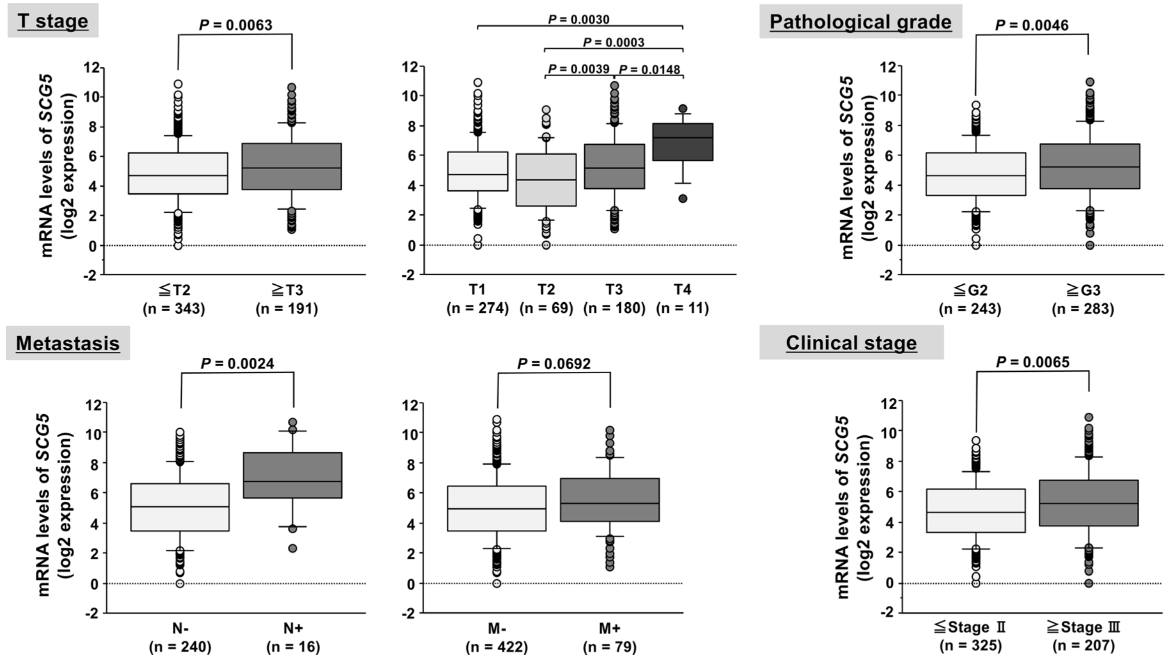

Supplementary Figure 6: Correlations between *SCG5* expression and clinicopathological parameters in TCGA ccRCC cohort. There were significant correlations between *SCG5* expression and several clinicopathological parameters (i.e., T stage, pathological grade, lymph node metastasis, and clinical stage). There was a trend but no significant correlation between *SCG5* expression and distant metastasis status.

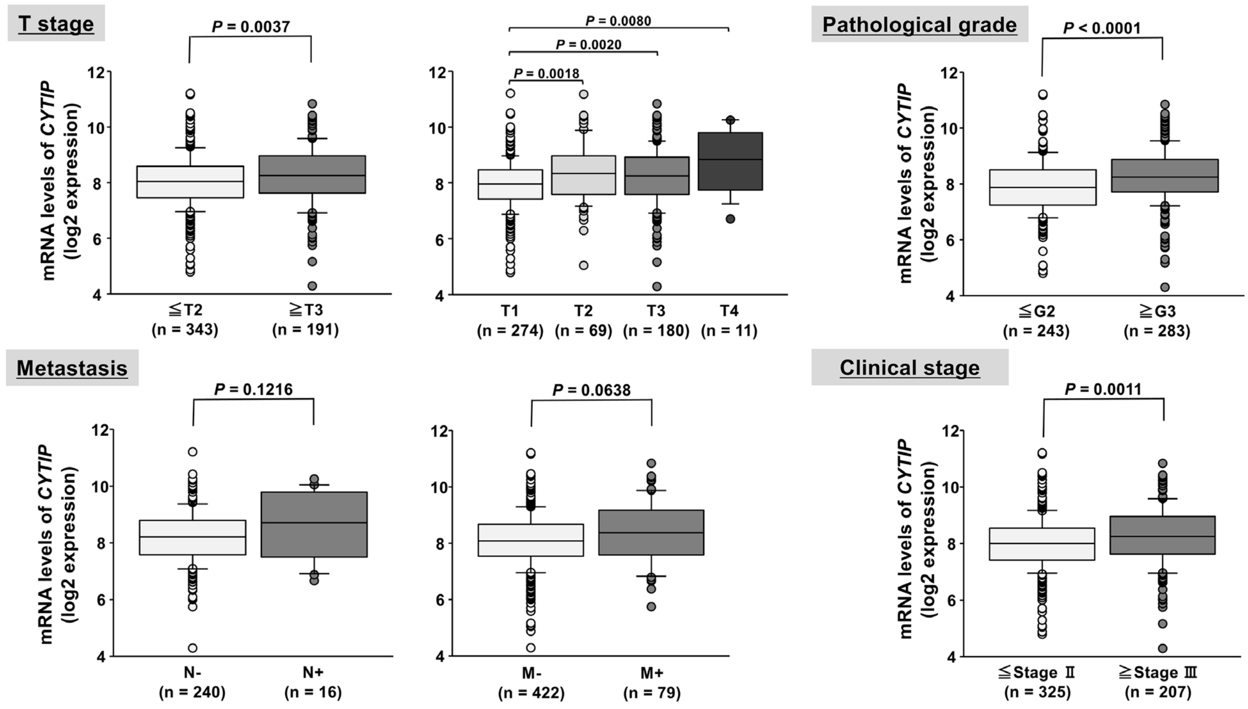

Supplementary Figure 7: Correlations between *CYTIP* expression and clinicopathological parameters in TCGA ccRCC cohort. There were significant correlations between *CYTIP* expression and several clinicopathological parameters (i.e., T stage, pathological grade, and clinical stage). There were trends but no significant correlations between *CYTIP* expression and lymph node or distant metastasis status.

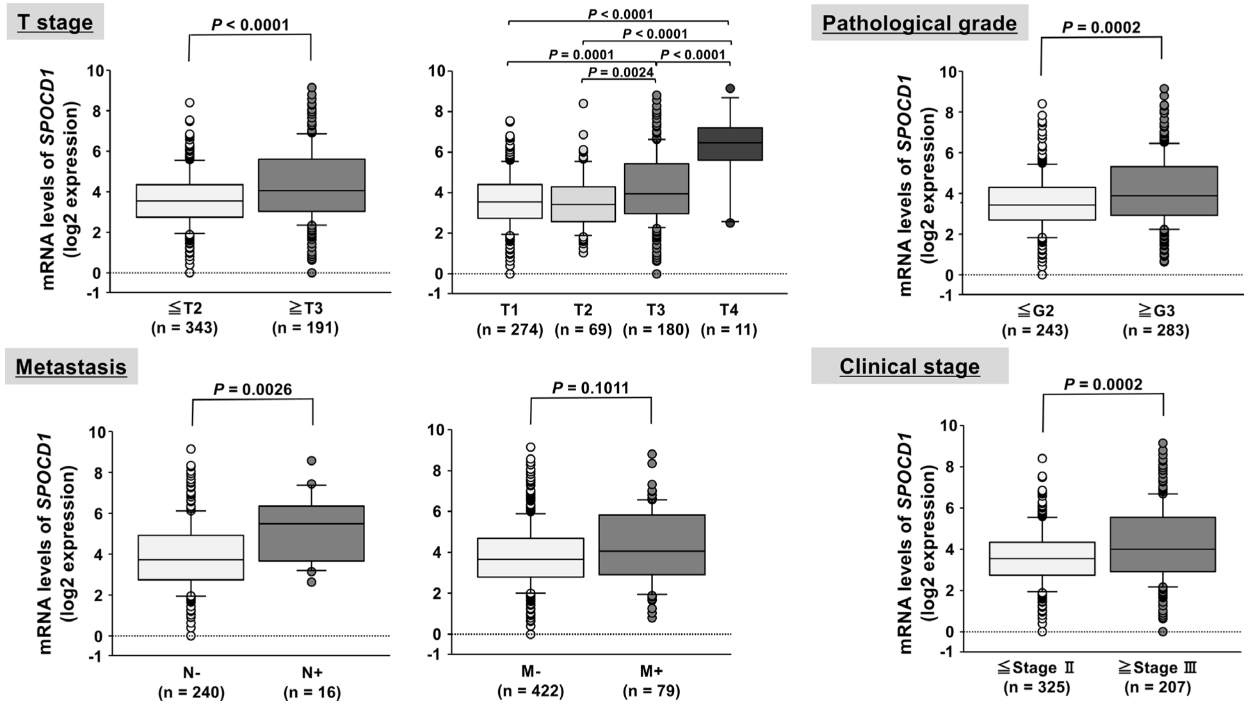

Supplementary Figure 8: Correlations between *SPOCD1* expression and clinicopathological parameters in TCGA ccRCC cohort. There were significant correlations between *SPOCD1* expression and several clinicopathological parameters (i.e., T stage, pathological grade, lymph node metastasis, and clinical stage). There was a trend but no significant correlation between *SPOCD1* expression and distant metastasis status.

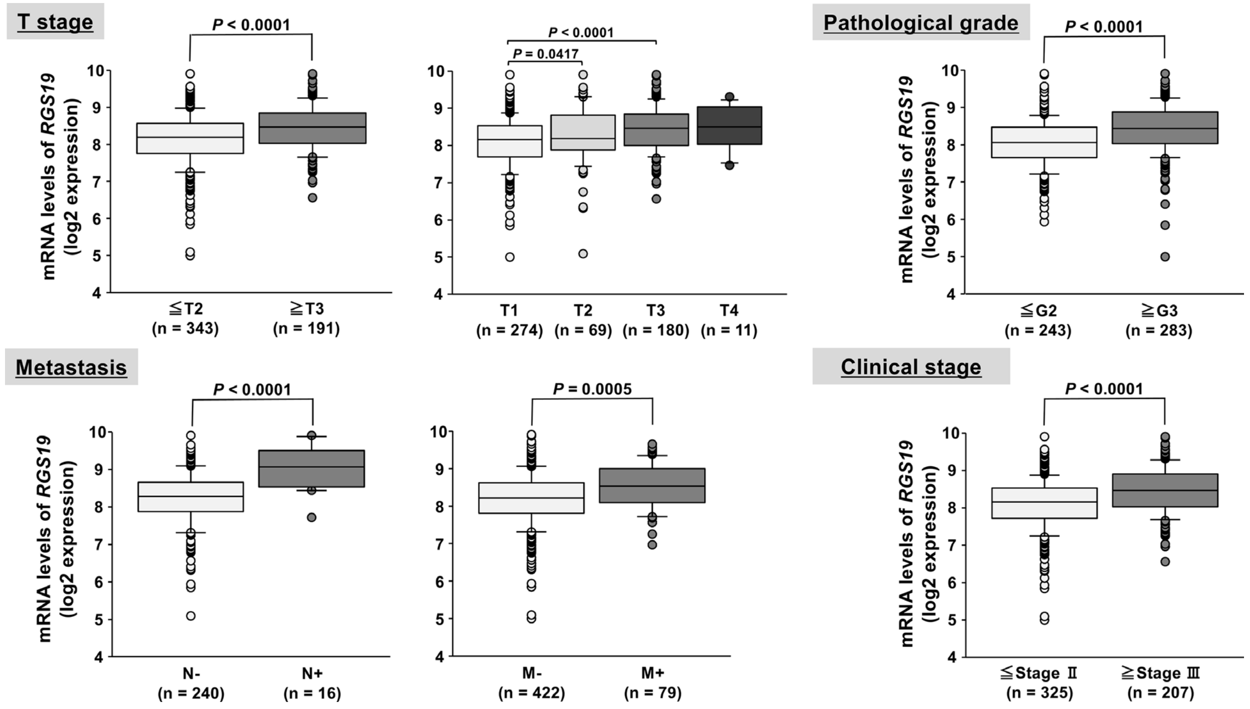

Supplementary Figure 9: Correlations between *RGS19* expression and clinicopathological parameters in TCGA ccRCC cohort. There were significant correlations between *RGS19* expression and several clinicopathological parameters (i.e., T stage, pathological grade, lymph node or distant metastasis, and clinical stage).

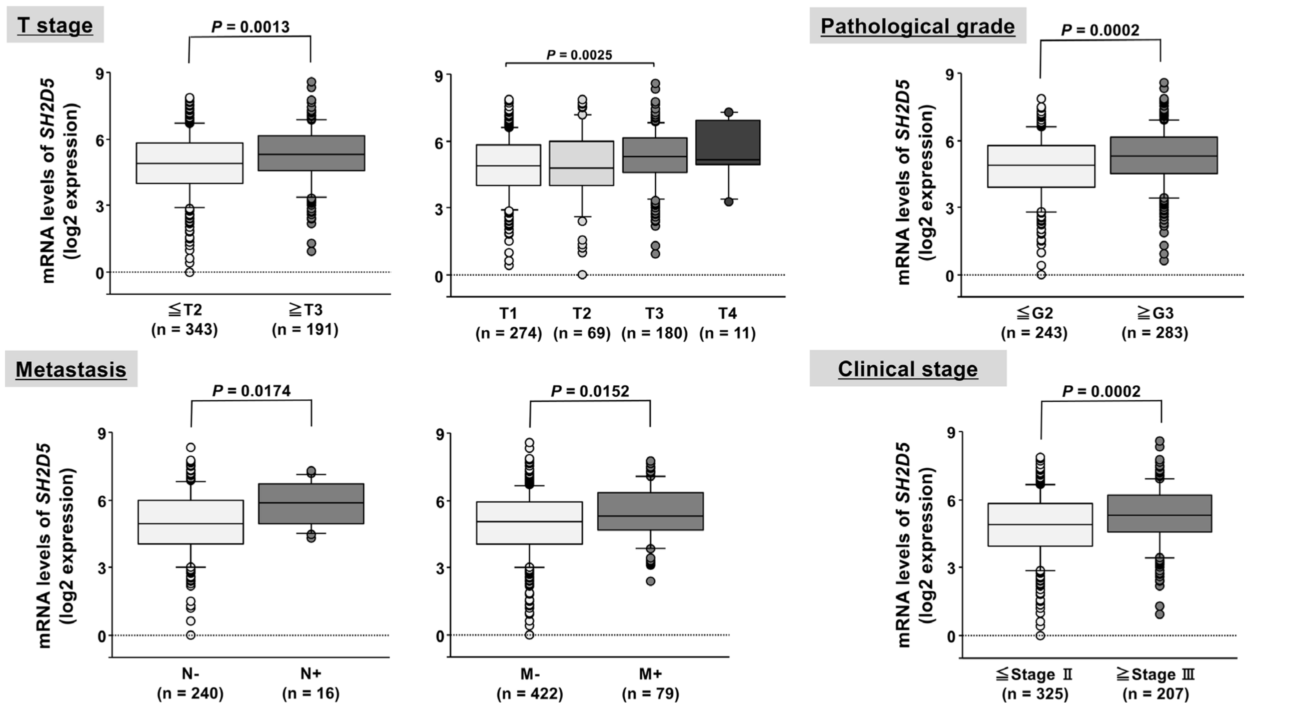

Supplementary Figure 10: Correlations between *SH2D5* expression and clinicopathological parameters in TCGA ccRCC cohort. There were significant correlations between *SH2D5* expression and several clinicopathological parameters (i.e., T stage, pathological grade, lymph node or distant metastasis, and clinical stage).

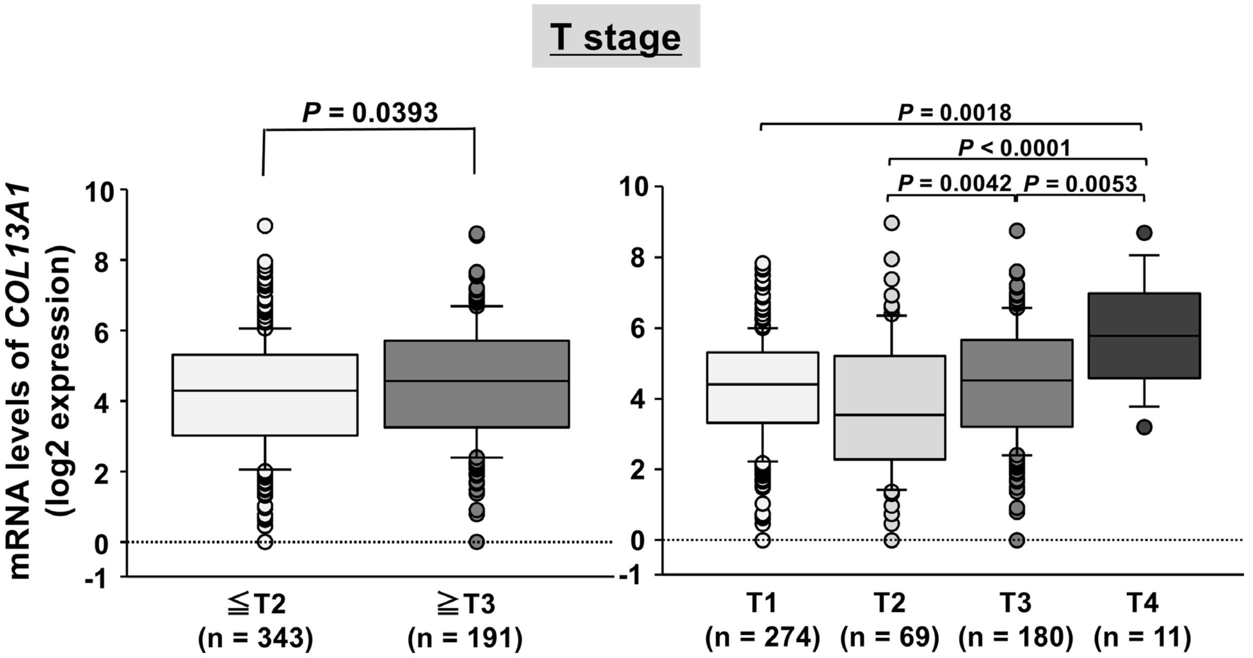

Supplementary Figure 11: Correlation between *COL13A1* expression and T stage in TCGA ccRCC cohort. There was a significant correlation between *COL13A1* expression and T stage status.

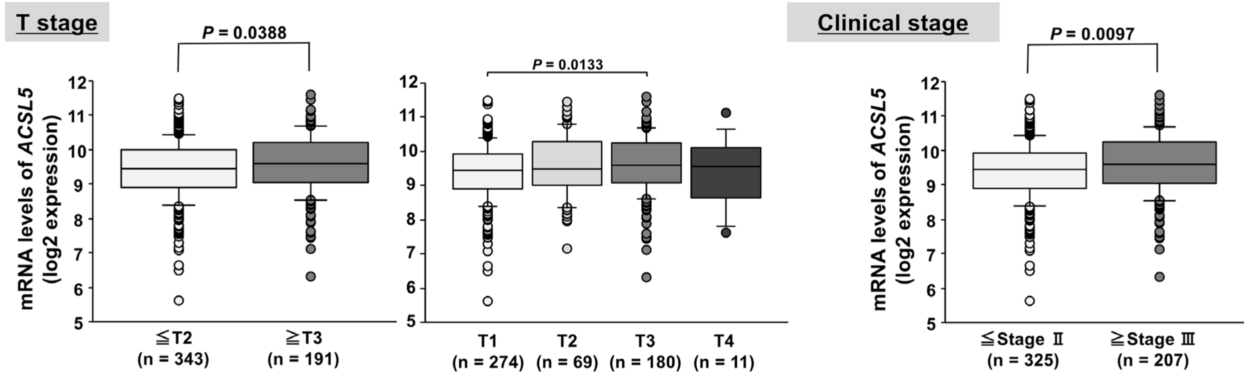

Supplementary Figure 12: Correlations between *ACSL5* expression and clinicopathological parameters in TCGA ccRCC cohort. There were significant correlations between *ACSL5* expression and several clinicopathological parameters (i.e., T stage and clinical stage).

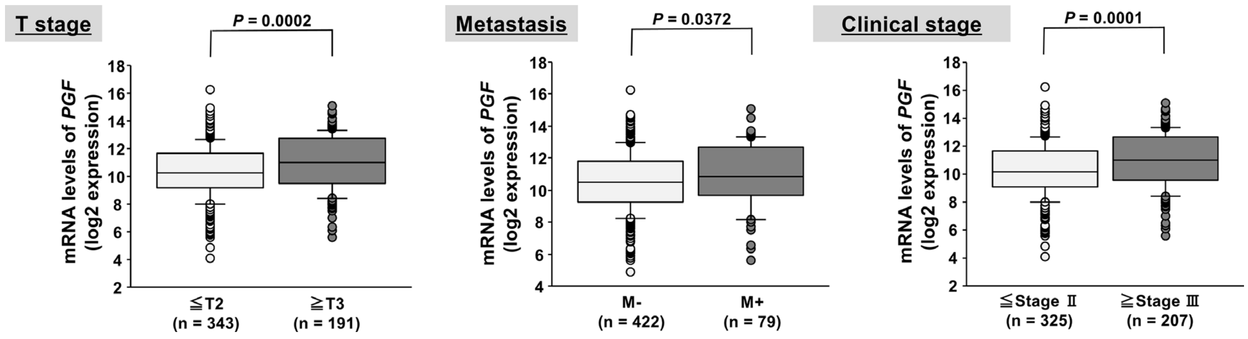

Supplementary Figure 13: Correlations between *PGF* expression and clinicopathological parameters in TCGA ccRCC cohort. There were significant correlations between *PGF* expression and several clinicopathological parameters (i.e., T stage, distant metastasis, and clinical stage).

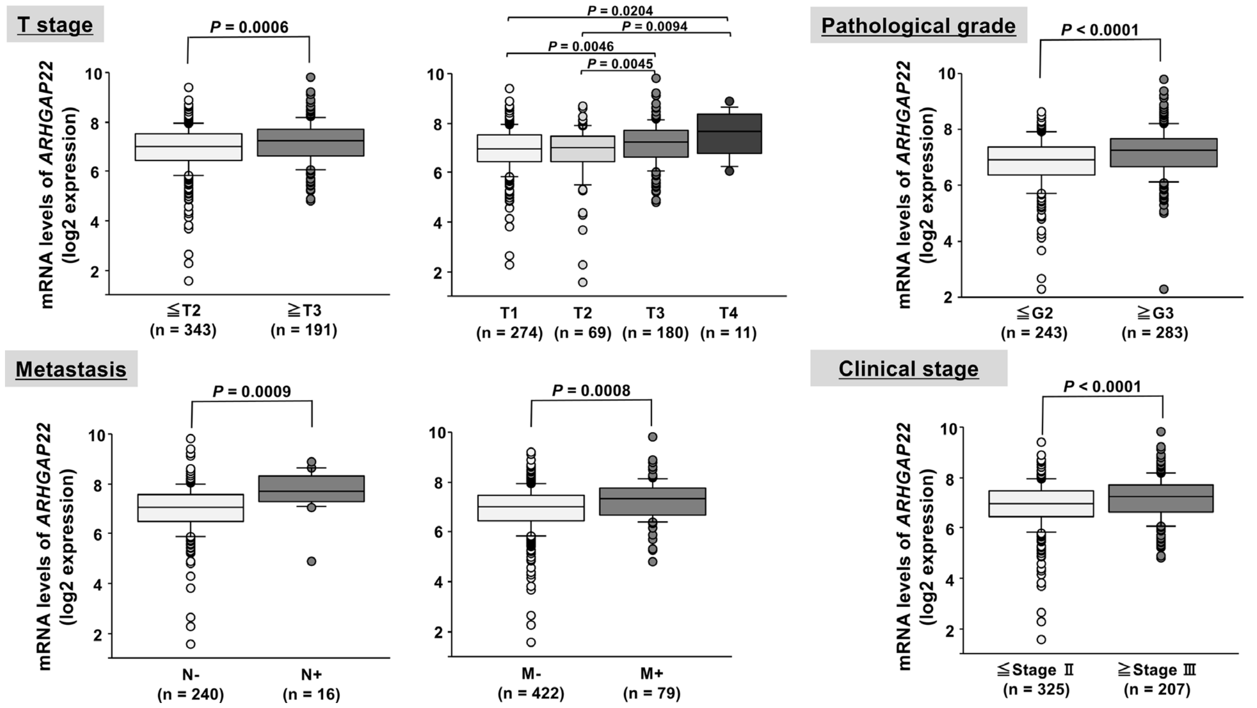

Supplementary Figure 14: Correlations between *ARHGAP22* expression and clinicopathological parameters in TCGA ccRCC cohort. There were significant correlations between *ARHGAP22* expression and several clinicopathological parameters (i.e., T stage, pathological grade, lymph node or distant metastasis, and clinical stage).

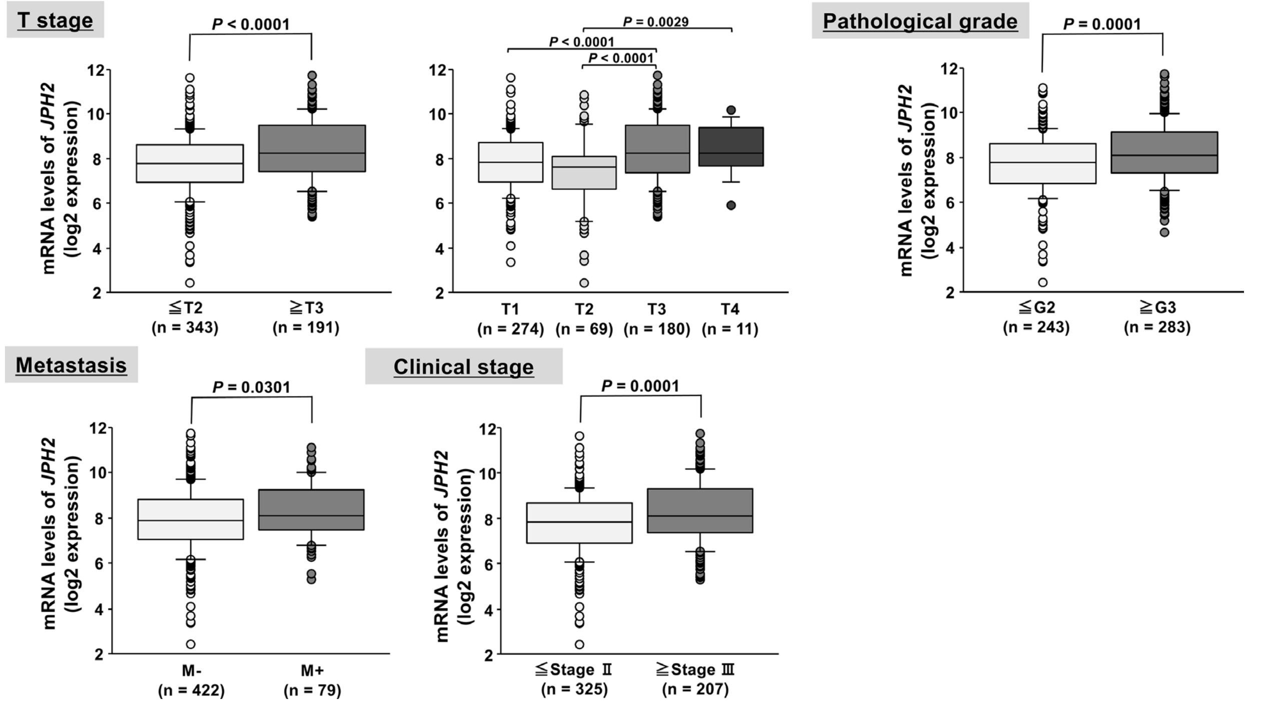

Supplementary Figure 15: Correlations between *JPH2* expression and clinicopathological parameters in TCGA ccRCC cohort. There were significant correlations between *JPH2* expression and several clinicopathological parameters (i.e., T stage, pathological grade, distant metastasis, and clinical stage).

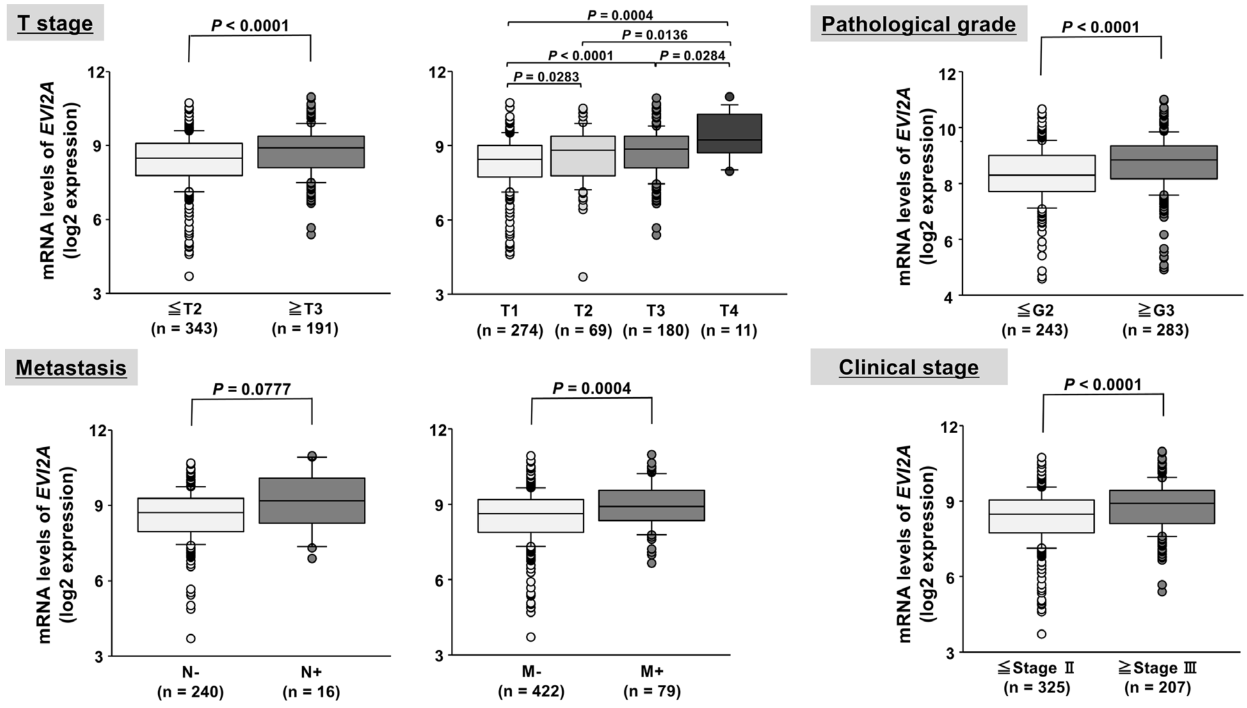

Supplementary Figure 16: Correlations between *EVI2A* expression and clinicopathological parameters in TCGA ccRCC cohort. There were significant correlations between *EVI2A* expression and several clinicopathological parameters (i.e., T stage, pathological grade, distant metastasis, and clinical stage). There was a trend but no significant correlation between *EVI2A* expression and lymph node metastasis status.

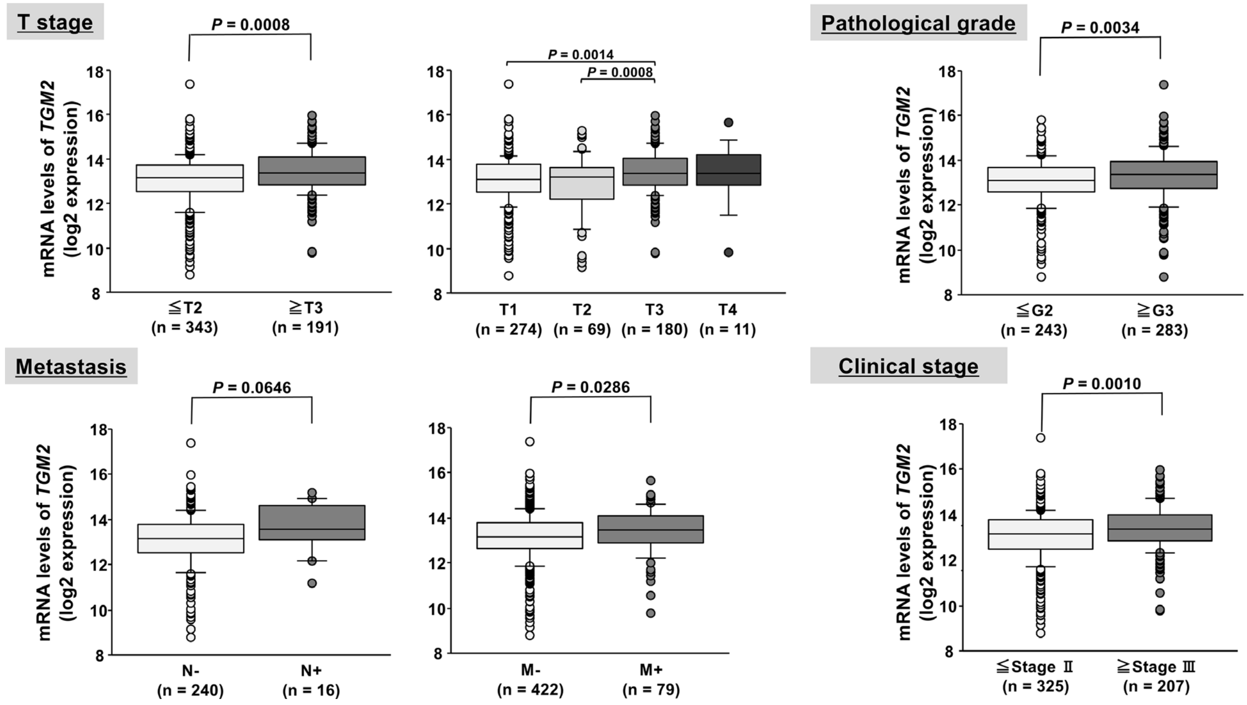

Supplementary Figure 17: Correlations between *TGM2* expression and clinicopathological parameters in TCGA ccRCC cohort. There were significant correlations between *TGM2* expression and several clinicopathological parameters (i.e., T stage, pathological grade, distant metastasis, and clinical stage). There was a trend but no significant correlation between *TGM2* expression and lymph node metastasis status.

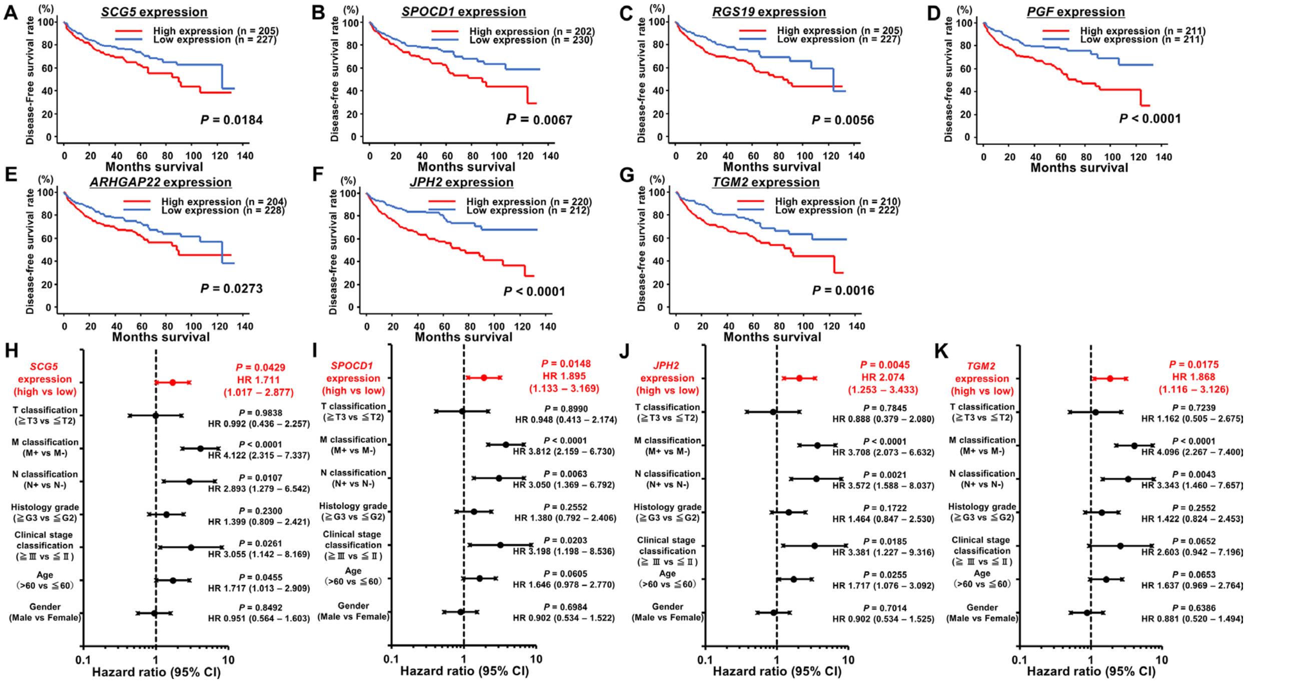

Supplementary Figure 18: Kaplan-Meier survival plots for disease-free survival in TCGA ccRCC cohort. (**A**–**G**) For 7 genes (*SCG5*, *SPOCD1*, *RGS19*, *PGF*, *ARHGAP22*, *JPH2*, and *TGM2*), disease-free survival periods were significantly shorter in patients with high mRNA expression than in patients with low expression in ccRCC. (**H**–**K**) Cox proportional analysis showed that *SCG5*, *SPOCD1*, *JPH2*, and *TGM2* were independent predictors of disease-free survival in ccRCC.
